# An experimental approach on dynamic occlusal fingerprint analysis to simulate use-wear localisation and development on stone tools

**DOI:** 10.1038/s41598-024-70265-1

**Published:** 2024-08-29

**Authors:** Hannah Rausch, João Marreiros, Ottmar Kullmer, Lisa Schunk, Walter Gneisinger, Ivan Calandra

**Affiliations:** 1 Department of Pre- and Protohistoric Archaeology, University of Münster, Münster, Germany; 2 Department of Human Origins, Max Planck Institute for Evolutionary Anthropology, Leipzig, Germany; 3 TraCEr, MONREPOS Archaeological Research Centre and Museum for Human Behavioural Evolution, LEIZA, Neuwied, Germany; 4 Institute for Prehistoric and Protohistoric Archaeology, Johannes Gutenberg University, Mainz, Germany; 5 ICArEHB, Interdisciplinary Center for Archaeology and the Evolution of Human Behaviour, University of Algarve, Faro, Portugal; 6 Senckenberg Research Institute and Natural History Museum, Frankfurt, Germany; 7 Institute of Ecology, Evolution, and Diversity, Goethe University, Frankfurt, Germany; 8 Faculty of Historical and Pedagogical Sciences, Institute of Archaeology, University of Wroclaw, Wrocław, Poland; 9 McDonald Institute for Archaeological Research, University of Cambridge, Cambridge, UK; 10 IMPALA, Imaging Platform at LEIZA, MONREPOS Archaeological Research Centre and Museum for Human Behavioural Evolution, LEIZA, Neuwied, Germany

**Keywords:** Archaeology, Archaeology

## Abstract

Information about the use of stone tools in the past is encoded in the wear patterns left on their surface; however, post-depositional processes can modify and obstruct these traces. One aim in the field of lithic functional analysis is to develop methods to detect and quantify these traces on stone tools. The occlusal fingerprint analysis (OFA) is a well-established method in dental wear studies to virtually simulate dental occlusal (contact between teeth) stroke movements and thus locate and quantify the sequential contact between opposing tooth crowns. Reaching across disciplines, we conducted controlled experiments to test the applicability of the OFA method on stone tools to address the challenge of use-wear quantification and localisation, and therefore the identification of post-depositional wear. Our findings reveal a clear overlap between zones of experimentally produced use-wear and OFA-calculated contact areas. We demonstrate OFA as a potential method to generate models of multiscale use-wear that can be used as references on experimental tools to identify post-depositional surface modifications on stone tool artefacts.

## Introduction

The field of lithic functional analysis is active in investigating the function, design, and handling of stone tool-based technologies of past humans. In functional analysis, use-wear analysis and experiments are combined to generate references to identify and infer the function of archaeological artefacts in question^1^. Techniques include the detection of residues and multiscale use-wear traces such as edge damage and polish on the surface of stone tools resulting from their use^2^. Detecting and quantifying these traces is crucial to accurately infer the function of stone tool artefacts^3,4^. However, the development of secure methods for the detection and quantification of use-wear traces is still in its infancy (but see^5–8^). Importantly, methods of use-wear localisation and quantification linked to specific actions would allow analysts to differentiate between human produced and post-depositional wear.

The challenge of identifying post-depositional wear was first addressed by Semenov^9^ who listed processes such as patination, abrasion by water- or wind-borne sediments that can affect the surface of archaeological artefacts. Building on this, Keeley^10^ listed the effects of soil movement to the use-wear on stone tool surfaces. Trampling is also known to cause alterations to the edge of tools resulting in edge damage and fractures^11^. These processes can modify or destroy the use-wear evidence on the surface of tools and thus obscure their identification^2^ and can even resemble traces generated by human use^12^. Therefore, differentiating between use-wear traces and post-depositional alterations is fundamental in making accurate inferences on the function of tools. While depositional factors are considered when investigating the function of stone tools, there is a lack of secure methodology for differentiating between these types of wear. Currently, the consensus is that wear randomly distributed on the surface of a tool or across the entire surface is considered post-depositional, while use-wear is located on the functional parts only^2^. Nevertheless, use-wear may be overseen or misinterpreted when post-depositional processes obstruct their visibility or mask the original contact areas between the tools and contact (i.e., worked) material. While some surface areas may have been functionally involved in the past, their use-wear may have since been overwritten or deleted beyond recognition (but see^13^). Likewise, other areas of use-wear may have remained largely unaffected by post-depositional processes.

Due to their robust nature, teeth tend to preserve relatively well in most soils and are therefore an important source of information for palaeontological, paleoanthropological and zooarchaeological studies. By studying the number of teeth in the mouth (the dental formula), the shape of the crowns, and in particular, specific patterns of erosion, abrasion, and attrition (i.e., dental wear), data is generated on vertebrate diet^14,15^. This is used to reconstruct food consumption habits^16^ and ultimately detect evolutionary changes in mastication^17^.

Dental wear has much in common with use-wear, as the formation of wear on the surface of teeth is based on similar tribological principles as those involved in the abrasion of stone tool surfaces (of course, different materials are involved). Both disciplines face similar methodological challenges such as those related to sample preparation, experimentation, observer errors, objective quantification and reproducibility; however, exchanging and applying methods across disciplines is rare (but see^18–21^).

An established method in dental wear studies is the Occlusal Fingerprint Analysis (OFA) method^22,23^. The software involves digital three-dimensional recordings, collision algorithm detections and imaging techniques to describe the contact of moving upper and lower dentitions, derived from virtual 3D models of teeth. Chewing movement of interacting 3D-modelled teeth are simulated to quantify the development of wear facets on dental surfaces.

The occlusal contact surface between the teeth of the upper and lower jaw are dynamic within the mechanical wear stages of a chewing-cycle^24^. In this context, fingerprint refers to the uniqueness of the occlusal wear pattern of an individual represented by wear facets on the teeth. While the position of such wear facets on molars is known for most mammals, their final appearance, including their detailed shape and size is highly variable and dependent on many factors such as age, environmental and behavioural factors as well as the morphology of teeth and the relationship of the antagonists^25–28^.

OFA has been applied to both fossilized and recent mammalian teeth^26,29–31^ to generate contact and performance data of chewing cycles. Palaeontologists and paleoanthropologists therefore study evolutionary trends of mammal dentition, function and evolution^16,25,29,31^.

Here, we applied the OFA method to a set of experimentally made and used stone tool samples. We assessed the applicability of OFA to address the challenge of use-wear localisation and quantification and therefore for the detection of post-depositional wear. First, we tested whether the contact areas simulated in OFA correspond to zones of use-wear traces observed on the tools’ surfaces through conventional use-wear analysis. While we considered both micro use-wear (i.e., surface polish) and macro use-wear (edge damage), we focused our analyses on micro use-wear traces. Second, we explored the possibility to use OFA to quantify use-wear on the stone tool samples. Lastly, we investigated how OFA may contribute to studying the performance of specific tool features and morphologies based on the generated contact areas.

## Results

### OFA and use-wear analysis

**Figure 1:**
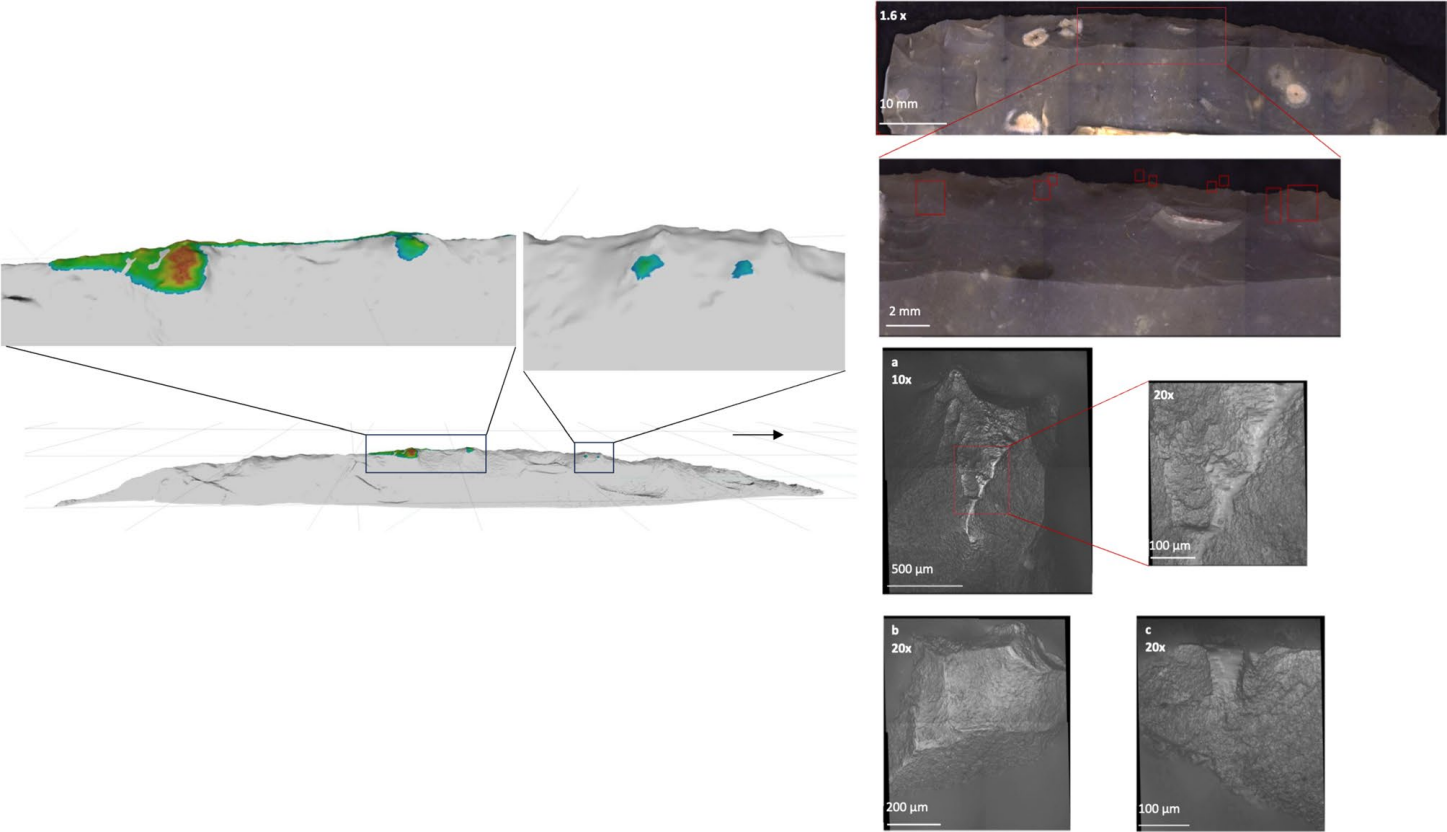
Left: OFA contact areas on the dorsal side of knapped sample FLT13-1 shown as a gradient from red (close) to blue (far). The arrow indicates the direction of the linear trajectory. Right: Micro use-wear (polish) identified in microscopic analysis after 2000 unilinear cutting strokes.

**Figure 2:**
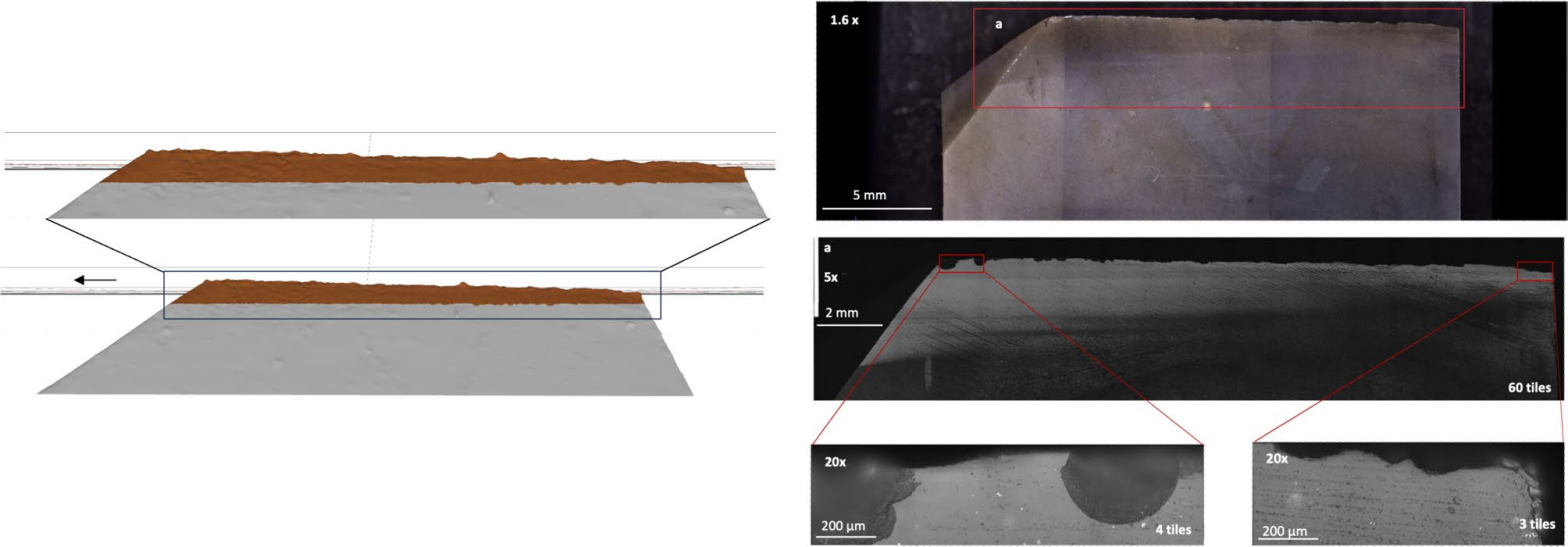
Left: OFA contact areas on the dorsal side of standard cut sample FLT8-13. The arrow indicates the direction of the linear trajectory. Right: Micro use-wear (polish) identified in microscopic analysis after 2000 unilinear cutting strokes.

OFA was used to simulate contact areas on four experimental tool samples and contact material sets. The OFA simulations generated contact on the widest parts and heightened topographies of the knapped samples (FLT13-12 and FLT13-1; Fig. 1, S1–3) on both faces of the edge, including the leading edge of the knapped sample tested on wood (FLT 13–12). For standard-cut samples, contact was calculated along both the ventral-, dorsal-, and the leading edges of the standard-cut sample on wood (FLT8-13; Fig. 2 and S4) and bone (FLT8-1; S5). In general, contact areas were larger for samples that abraded the wood contact material compared to the synthetic bone; contact areas were also larger on the standard-cut samples compared to the knapped tools (Table 1). A trend common across all simulations was the larger contact area on the contact material compared to the sample; the ventral side of samples also had more extensive contact than the dorsal side. For the knapped samples, the maximum contact area size calculated in OFA ranged between 8.61 and 9.26 mm^2^ (Table 1, S22, S23). For the standard-cut samples this ranged between 11.91 and 27.08 mm^2^ (Table 1, S24, S25). Contact area sizes were based on the moment of largest contact; it is not a cumulative value of the entire stroke trajectory.

**Table 1:** Size of micro use-wear contact area measured in ZENConnect compared to contact area sizes calculated in OFA.

Sample set	Sample	Size of micro use-wear contact area [mm^2^] measured in ZENConnect	Contact area size [mm^2^] at moment of maximum contact calculated in OFA
Set 1: Knapped sample FLT13-12 on wood OFA-WP1	FLT13-12	7.85	9.26
Set 2: Knapped sample FLT13-1 on bone OFA-BP1	FLT13-1	0.56	8.61
Set 3: Standard cut sample FLT8-13 on wood OFA-WP1	FLT8-13	39.25	27.08
Set 4: Standard cut sample FLT8-1 on bone BP-cutting	FLT8-1	4.13	11.91

Overlap in terms of contact area size between measured micro use-wear polish and OFA calculations was poor (Table 1): The contact area sizes calculated in OFA were larger than the measured micro use-wear in all cases but set 3 (standard-cut tool sample on wood). While the edge length of each sample was different (the knapped samples had longer edges), the affected surface area was nonetheless consistently smaller for the knapped samples, as expected.

Locations of macro (i.e., edge damage) and micro (i.e., polish) use-wear were compared to the locations of contact generated in OFA. Like in OFA simulations, use-wear was observed on the surface of the widest parts and highest topographies of the knapped samples (Fig. 1 and S6–S15). The most developed micro use-wear was found towards the leading edge of samples tested on wood (FLT13-12, S9; FLT8-13, S17). Use-wear was located along the whole edge of the standard-cut sample tested on wood and discontinuously on the standard-cut sample tested on bone (Fig. 2 and S21–S22). In sum, based on visual inspections, zones of use-wear overlapped with the calculated OFA areas. The use-wear on samples tested on wood (FLT13-12, S17–S19; FLT8-13, S7–S10) had the highest degree of overlap with the OFA calculations. On visual inspection, the OFA contacts for the samples tested on bone (FLT13-1, S11–S16; FLT8-1, S20–S22) were larger than use-wear areas.

### Experimental data

Sensor data from the material tester (SMARTTESTER) was studied as a second level of comparison to the OFA software results. The material tester was set to perform 2000 unilinear strokes for each experimental setup. The actual number of performed strokes deviated from this (Table 2, S27–S30) likely due to software issues.

**Table 2:** Number of unilinear cutting strokes programmed vs. actual number performed with the SMARTTESTER.

Sample set	Programmed number of strokes	Number of strokes performed
Set 1: Knapped sample FLT13-12 on wood OFA-WP1	2000	1999
Set 2: Knapped sample FLT13-1 on bone OFA-BP1	2000	2249
Set 3: Standard cut sample FLT8-13 on wood OFA-WP1	2000	1999
Set 4: Standard sample FLT8-1 on bone BP-cutting	2000	2000

Recording velocity and force enabled us to check the degree of control during the experiments. Velocity was comparable across all experiments and reached the input value of 600 mm/s at approximately 105 mm of the cutting stroke for each sample set (S27–S30). Force was variable within each experiment and across all experimental sets. The exception was FLT8-1/BP-cutting^32^, which shows consistency within the experiment itself. While the target force was applied via 5 kg dead weights (approximately 50 N), force originated at approximately 60 N for all experiments.

Moments of increased friction corresponded to moments of increased contact areas calculated in OFA, supporting the data generated in OFA simulations.

**Figure 3:**
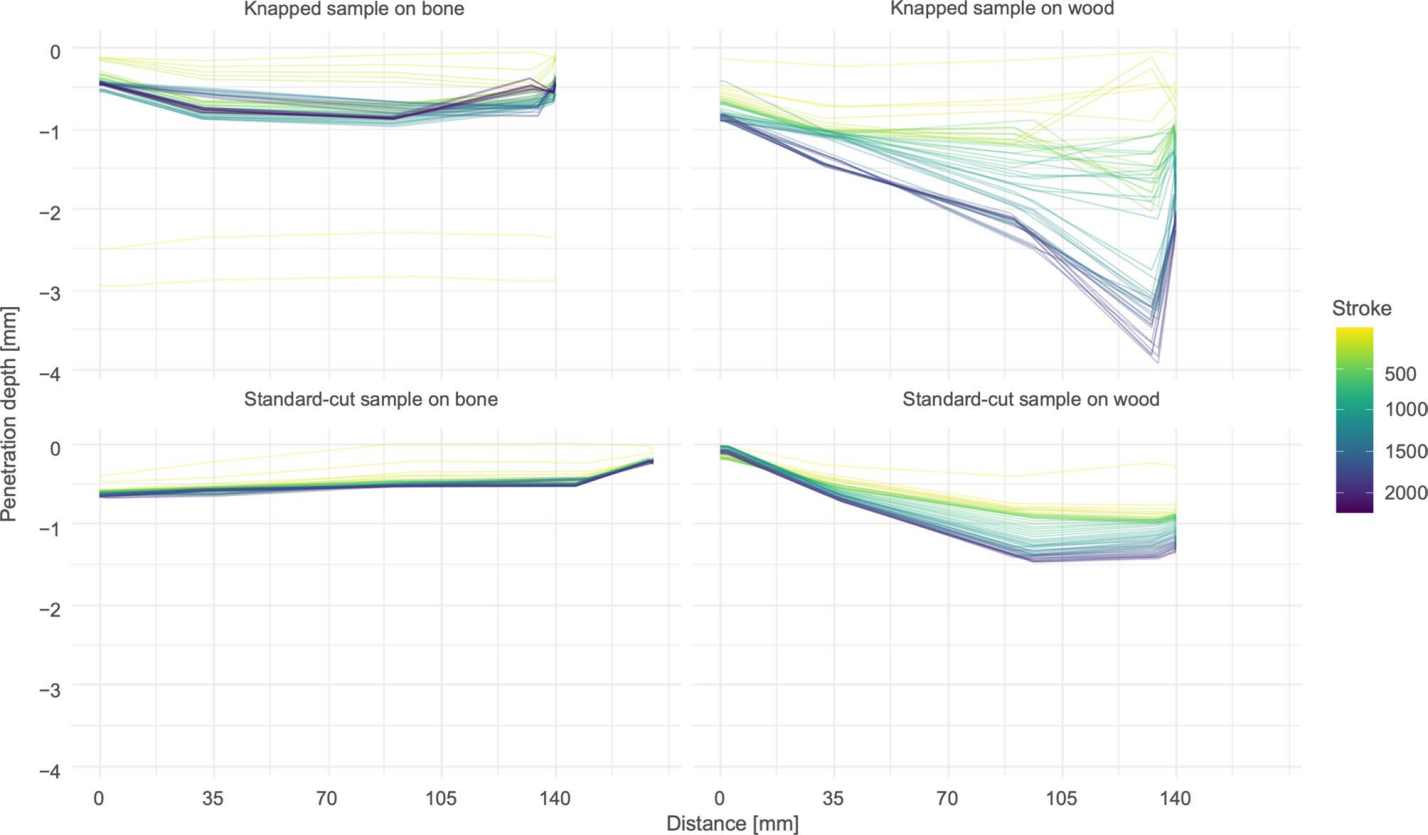
Penetration depth of samples in contact material throughout experimentation. Distance is the position on the contact material from the origin of each stroke. Lines illustrate every 40th cutting stroke of the whole experiment from yellow to purple.

Last, samples tested on wood had deeper penetrations than those tested on synthetic bone. Knapped samples reached deeper penetrations compared to standard-cut samples cutting the same material (Fig. 3).

## Discussion

The results of our study demonstrate that the location of OFA contact areas match recorded zones of use-wear (specifically micro use-wear) on experimental samples. OFA contact areas were generally larger than the observed micro use-wear (Table 1), suggesting that OFA may detect zones on the surface of experimental stone tools where use-wear is most likely to develop.

**Figure 4:**
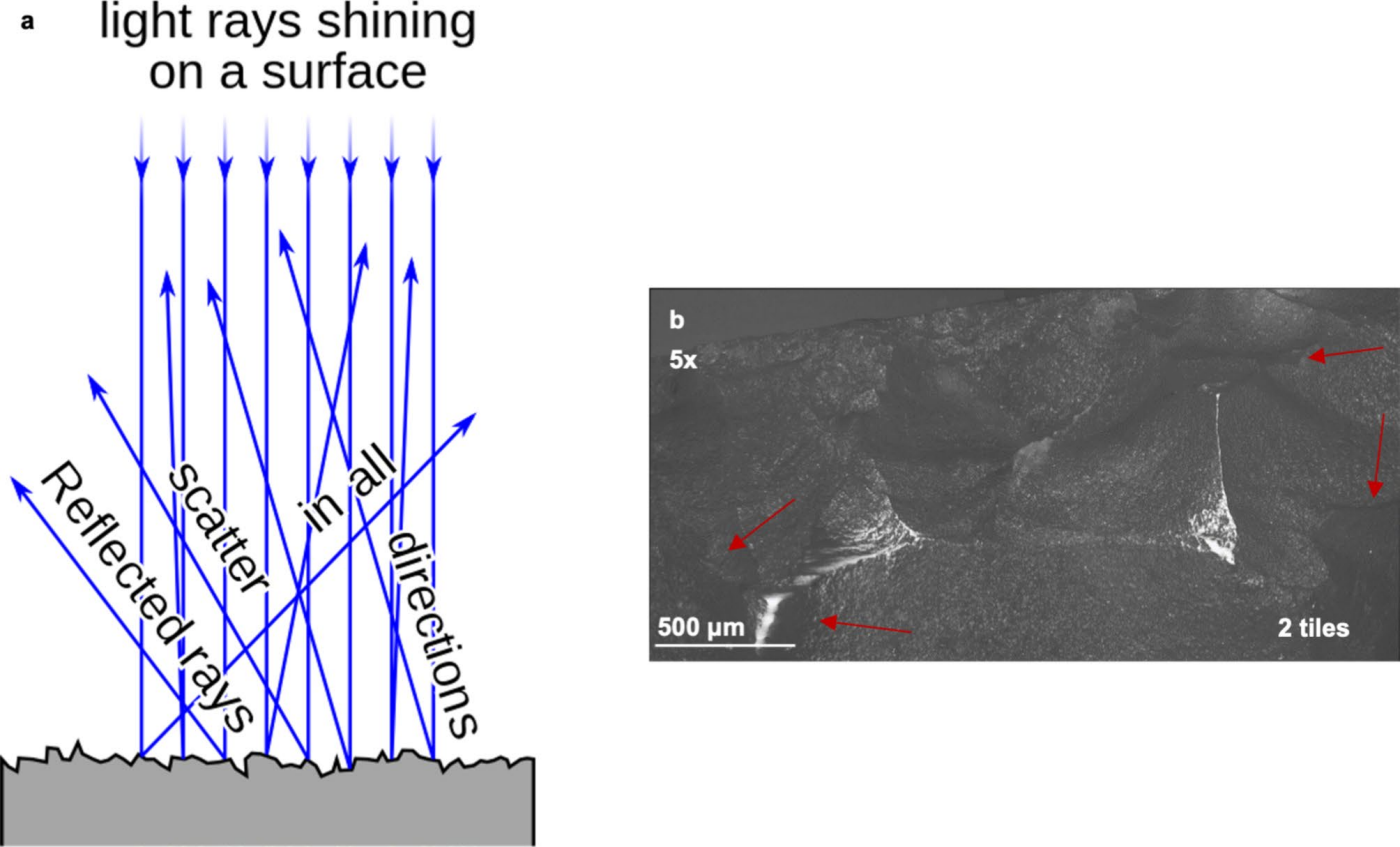
(**a**) Schematic depiction of the law of reflected light, specular and diffuse reflection^33^. (**b**) EDF-stitched image of polish on knapped tool sample FLT13-12 with examples of diffuse reflection (red arrows).

Alternatively, or complementarily, this discrepancy can be due to the fact that OFA assesses surface areas on 3D-models (OFA) rather than 2D images (micro use-wear): 3D areas are always larger than projected areas. The limited visibility of micro use-wear during microscopy caused by diffuse light (Fig. 4) likely also contributed. Micro use-wear on the surface of a sample was identified during microscopy by characteristic bright areas caused by light reflected from the abraded surface. Therefore, the position of the sample and the angle of its surface to the microscope light is decisive as to which areas are visible during microscopy. Last, the OFA contact areas were based on the moment of highest contact across the digital trajectory (S22-S25) and not the cumulative contact of the entire stroke. If the contact areas were cumulative, they would be even larger.

For the standard cut sample on wood (FLT13-12) however, the calculated polished area (39.55 mm^2^) was larger than the OFA contact area (27.08 mm^2^). We attribute this discrepancy to an interplay of factors related to the limits of the OFA software, contact material properties and tool morphology. The OFA software was developed to generate contact areas between interacting 3D models without the input of kinetic data. In the experiments performed here, however, force was applied to the tool samples (5 kg) to generate an abrasive penetration of the contact material. The experimental stroke is simulated in OFA without consideration of these acting (vertical) forces, meaning that the tool sample may have penetrated the contact material deeper than simulated in OFA. This would result in a smaller OFA contact area on the tool sample compared to the measured polish. The softer property of the wood as a contact material (see below) and the low angle of the tool's unretouched edge likely exacerbated this because the tool sample penetrated the contact material deeper with a lower amount of force. This is corroborated by the higher penetration depth of the tool samples on wood as compared to bone (Fig. 3).

In general, samples abrading wood (FLT13-12, FLT8-13) had larger use-wear and OFA contact areas compared to samples abrading bone (FLT13-1, FLT8-1). We suggest this is related to the material properties of these contact materials. The pinewood slab used is a softwood with a lightweight cellular structure that has a low density^34^. The wood is structurally damaged with less force or is damaged more severely using the same amount of force compared to synthetic bone. Further, by avoiding knots and arranging the cutting strokes on the tangential surface along the growth rings, there was less resistance to structural damage than if the strokes were positioned across the growth rings. Therefore, samples on wood penetrated deeper (S27–S30), resulting in larger contact areas. Furthermore, in comparison to the bone contact material from which dislodged particles were quickly removed from between the cutting groove and the tool sample, fibres from the wood contact material remained intact with the wood slab for a longer period of time. This suggests that the tool samples abrading wood were in contact with and abrading a larger surface area compared to the bone contact material. It has been demonstrated that soft contact materials such as wood cause smoother abraded surfaces than with harder material such as bone^4^. Our results demonstrate that there is a correlation between the nature of the contact material and the size of the resulting micro use-wear area, with soft materials correlating to larger polished areas.

Sensor data delivered information on the level of control during and across experiments. It was also used as a second level of control for the OFA simulations since we expect larger contact areas to result in increased friction, for a given force applied. Velocity was well controlled (i.e., followed a similar trend for each experimental stroke, S27–S30) and was comparable across all experiments. There was high variation in force, both within each experimental set and across sets. Although a target force of ~ 50 N was set for each sample (using 5 kg dead weights), each experiment began with ~ 60 N according to the force sensor readings. This discrepancy was likely due to the export of system readings rather than tare weight readings, which would have compensated the weight of the dead-weight frame plus tool sample (approximately 1 kg ~ 10 N). In addition, we cannot exclude that the force spring supposed to nullify the weight of the dead-weight frame and sample was not precisely calibrated, resulting in a force applied to the sample potentially slightly different from the target ~ 50 N. The stick–slip effect may have possibly amplified a potential spring calibration issue. We suggest stick–slip occurred in all experiments and was most distinct at the onset and end of the strokes at moments of change in velocity (S27–S30). Microscopically, stick–slip is related to the roughness of interacting surfaces with rougher surfaces requiring higher driving force for sliding motions to be initiated^35^. While smoother surfaces cause less friction, most surfaces have some degree of roughness as they are covered with ridges, pits, and scratches even if they appear flat^35^. During stick–slip, the asperities on the interacting surfaces interlock until the force is high enough to break them or slide over one another^35^.

**Figure 5:**
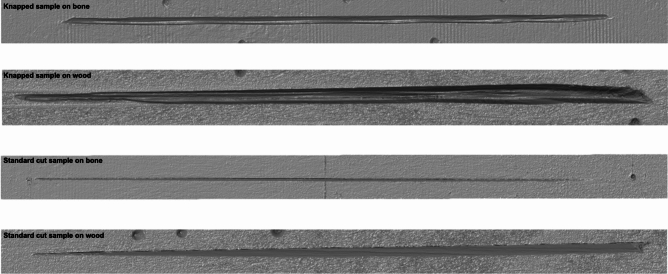
3D model images of the abraded contact materials. Images are not to scale. All grooves are 140 mm long, except for standard cut sample on bone which is 170 mm long.

Increases in friction were correlated with increases in OFA contact areas (S22–25, S27-S03), supporting the OFA predictions. The profile of the cutting grooves (Fig. 5) likewise correlate with the trends for friction (S27–S30), with increased friction from 35 mm onwards associated with increased depth in the cutting groove.

Penetration depth of tool samples in contact material delivers data on sample efficacy which can be compared across sample sets. We adopt a definition of efficacy (synonym to effectiveness) as the relationship between a goal and achievement^36^, in this case the cutting depth. Samples that worked wood (FLT8-13, FLT13-12) had relatively deeper penetrations due to the softer (i.e., lower hardness value) physical properties of wood in contrast to those of bone. Knapped samples reached deeper penetrations than the standard-cut samples on the same contact material. Therefore, it may seem surprising at first that for these samples, the contact areas (both measured and in OFA) were smaller than for standard-cut samples. We suggest this is related to surface topography. Standard-cut samples have a flatter topography, meaning that more surface area is in contact with the contact material. On the other hand, the knapped samples are more effective in cutting the contact material because their heightened topographic features (from retouch) caused a higher rate of abrasion. They reach deeper penetrations with the same number of cutting strokes. In these cases, due to the topography from retouch, the same amount of force is distributed across a smaller area of the tool compared to the standard-cut samples, where force is distributed across the whole edge.

In terms of performance, the penetration depth graphs also suggest that the standard-cut samples cut the contact material throughout the experiment more consistently compared to the knapped samples, meaning that a similar volume of contact material was abraded with each stroke (Fig. 3). We suggest this is related to the geometry of the edge and surface topography. A simpler edge geometry (i.e., fewer angles and faces) and a flatter surface topography (i.e., fewer hills and valleys), as the case of the standard-cut samples, causes a similar surface area to be in contact with the contact material across strokes. On the other hand, a complex geometry and rougher surface topography means that contact areas between the edge and the contact material is dynamic throughout the abrasion process. This was the case for the knapped blade samples.

To ensure a proper alignment between each experimental sample and its contact material, 3D scans of the relative position of these objects in the experimental setup were carried out (see “Materials and methods” section). The relative position scans were needed to align the high-resolution 3D scans of each sample and contact material which was essential for simulating a trajectory in OFA that was identical to the experimental setup. At the beginning of our study, the 3D scans of the relative position after experimentation were generated with a low-resolution 3D light-structured scanner (HP Structured Light Scanner Pro S2). The advantage of this scanner was its lightweight and flexibility to move around the large SMARTTESTER and attached cables. However, the resolution of these scans was not sufficient to properly align with the high-resolution 3D scans of each sample tool and contact material (made with the Aicon smartSCAN). Using the higher-resolution 3D scanner (Aicon smartSCAN) also for the relative position scans greatly improved the alignment. The disadvantage was however the bulky nature of the stand which did not allow for a complete rotation around the experimental setup of the sample and the contact material in the SMARTTESTER. In future work, if the alignment of samples and contact material in experimentation are precisely protocolled, this data can be used to directly align the 3D models in a 3D processing software or in OFA. In this case, scanning the relative position of the experimental setup may be omitted. This was demonstrated in our experiments through sample FLT8-1 and BP-cutting^37^ for which no relative position scan existed but the position of the sample and contact material was known. Even without a relative position scan, the overlap between OFA contact areas corresponded with use-wear zones.

## Conclusion

Our study demonstrates the potential of applying OFA to address specific questions related to stone tool assemblages in the experimental workflow. Controlled experiments can be used to test variables such as performed action, tool morphology and design, contact or raw material, and duration of use. Experimental trajectories can be reconstructed virtually in OFA and contact areas corresponding to use-wear generated. The localisation of use-wear zones can therefore be linked to a specific variable change. Our proposed method demonstrates potential for detecting use-wear development and changes over time when sequential experiments are performed. In simulating use-wear development and localisation, the contact areas generated in OFA can serve as proxies for studying artefacts from the archaeological record and for ruling out post-depositional wear.

We propose a twofold application of OFA in lithic functional analysis. First, it can be used to generate use-wear zone libraries linked to experimented variables (e.g., movement, contact material, tool morphology). For example, if wear is observed in a region where no contact should have occurred according to OFA reference simulations, it is likely that this wear is unrelated to use, regardless of its appearance. In this way, the spectrum of use-wear on the surface of experimental tools linked to specific variables can be narrowed. Second, OFA can be used to address specific hypothesis of tool performance by calculating surface areas involved in the abrasion process. While these inferences are preliminary here, they highlight the potential of the software for addressing questions related to use-wear development, tool performance such as effectiveness and further, on human decisions such as those relating to raw material and tool design such as retouch.

## Materials and methods

To address the research aim of applying OFA to experimentally produced and used stone tools, we developed a series of mechanised cutting experiments with four experimental sample sets. The variables of tool morphology and contact (i.e., worked) material were tested with experimental trajectory simulation in OFA.

A detailed description of the workflow corresponding to the materials and methods is available on protocols.io (see Data availability).

### Samples and contact materials

Four sample sets were prepared, each containing a tool sample and contact material sample. To reduce variability between the raw material of samples, all samples were made with Baltic flint from the same secondary deposit in Sweden. The variables of tool morphology and contact material were tested with experimental trajectory simulation in OFA. Two tool samples had a standard-cut morphology, while the other two were knapped into blades and laterally retouched. For the standard-cut samples, the raw material was sawed into rectangular cuboids (i.e., blanks) with a width of 3 cm. The end of each blank was modified again using a diamond bandsaw to produce an edge angle of 45°. The leading edge of the sample was chamfered at 45° to disperse the force across the entire edge during experimentation and to avoid immediate catastrophic fracturing of the edge during the first cutting strokes. The first standard-cut sample (FLT8-1) was used in a previous experiment with the same variables and controls, however cutting length varied (Schunk et al.^37^, see below). The second standard-cut sample (FLT8-13) was made by sawing off the base of sample FLT8-1. Again, the leading edge was chamfered at 45° in the same way as FLT8-1. One tool sample of each type was used on a synthetic (polyurethane) bone plate (SYNBONE), while the other was used to cut a softwood slab (*Pinus sylvestris*).

For standardisation and relocating positions on the sample between different microscopes, a coordinate system based on 100–200 μm diameter ceramic beads^38^ was adhered to the dorsal and ventral side of each sample with epoxy resin.

### Controlled experiments

A total of 2000 controlled unilinear cutting motions were performed with each sample set using the modular material tester SMARTTESTER^39^. Note that one knapped sample (FLT13-1) was originally tested sequentially (i.e., 4 rounds of increasing stroke number: 1–50; 51–250; 251–1000; 1001–2000). The original documentation workflow with a low-resolution 3D scanner was not sufficient to perform the OFA analysis at each cycle (see below).

Acceleration (target 4000 mm/s^2^), velocity (target 600 mm/s), force (5 kg dead weights), and the length of the cutting motion (140 mm) remained identical across all experiments, with the exception of the sample set 4 where the standard-cut sample (FLT8-1) travelled for a total of 170 mm^37^. In addition to these parameters, the penetration depth of the samples and friction between it and the contact material were recorded by sensors on the material tester. During experimentation, abraded contact material residues were removed by hand with an oil-free air compressor every 50 strokes to reduce lubrication/abrasion between residues and the samples.

After experimentation, a 3D scan was taken of the relative position of the sample and contact material in the experimental setup using the 3D structured light scanner AICON smartSCAN-HE R8. During the first round of experiments for sample FLT13-1, an HP Structured Light Scanner Pro S2 was used for this step. Since the resolution of the scan from this scanner was too poor, the AICON was selected for scanning. This relative position scan was not acquired for sample FLT8-1 as it was part of a previous experiment^37^. It is important to note that this alignment corresponds to the state at the end of the experiment, after the 2000th stroke (but see Table 2 for the performed number of strokes for each experiment). Therefore, it does not reflect the relative positioning during all strokes of the experiment. 3D scanning the experimental setup was necessary for acquiring a proper alignment of the 3D models of the experimented objects of each sample set in order to define in OFA an identical trajectory to that of experimentation.

### Cleaning protocol

After preparation, and again after experimentation, each sample was cleaned to remove residues of the contact material. A cleaning solution of 4 L of tap water with 4 mL of surfactant (BASF Plurafac LF901, 1 g/L = 0.1% w/v) was added to an ultrasonic bath for 5 min at 35 kHz and 40 °C. To remove any absorbed detergents, the samples were rinsed with distilled water. The edge of each sample was dried with acetone (≥ 99.5%) applied with cotton swabs. Fibres from the swabs were removed with an oil-free air compressor. Each cleaned sample was then screened under a stereomicroscope at 12.5× optical magnification to ensure that all residues were removed.

### Documentation

Before and after experimentation (in both cases after cleaning), each sample was imaged with a digital camera (D610 Nikon digital camera) to generate reference photos. High-resolution 3D scans of the samples (before and after experimentation) and contact materials (only after experimentation) were generated with the small field of view (S-150: 110 × 80 × 70 mm measuring volume) on the AICON smartSCAN structured light scanner in order to locate and quantify macro-wear (comparison before and after experiment), as well as to generate 3D models for OFA. One sample (FLT13-12) required additional coating with talcum powder (DM babylove sensitive Puder) to avoid the presence of large holes in the 3D mesh. An additional round of the cleaning protocol was therefore applied for this sample after 3D scanning. The contact material was scanned using the contour matching function and the samples were scanned using the automatic function with the turntable. The scans were merged using the OptoCat software.

Using a digital microscope (Zeiss Smartzoom 5) equipped with the 1.6×/0.1 objective, stitched, extended depth of focus (EDF) images of the dorsal and ventral side of the samples were acquired as overview photos, on which images of polish were later superimposed for localisation.

This was followed by micro-wear (here, polish) documentation using an upright light microscope (Zeiss Axio Imager.Z2). We identified polish by its dull, brighter or smoother character, following conventions in the field^10,40^. The images acquired in this step were later used to measure areas of polish to compare with the OFA calculations. The location of the polish on the edge was calibrated by using the coordinate beads, when possible with the 10×/0.4 objective, and later superimposed on the digital microscope overview images. The retouched blades were positioned with a large tilt. This caused the working distance (5.4 mm) of the 10× objective to be too tight, meaning that the 5×/0.2 objective (working distance = 21 mm) was used for calibration in some cases (dorsal sides of FLT13-1 and FLT13-12). For one sample in particular (FLT13-1), this led to a distorted overview image relative to the microscope images. Both the dorsal and ventral sides of samples were documented, as well as the leading chamfered edge of the standard-cut samples. Continuous areas of polish were imaged with a low-magnification objective (5× or 10× objective) to acquire an image of the overall area. Each area was sampled with the 20×/0.7 objective to acquire a detailed image of each polish.

Silicone moulds for short- and long-term storage of the edge of samples including the coordinate beads were made before and after experimentation. The silicone AccuTrans AB forensic silicone (polyvinylsiloxane) was used as it has been demonstrated well suited for archaeological samples^41^.

### Image and 3D processing

After documentation, all images acquired from the microscopes were processed using the ZENConnect software add-on for ZEN desk, ZEISS (v3.5 HF 9). Using the calibrated coordinate beads, the images of polish acquired with the upright light microscope were superimposed on the overview images from the digital microscope. In this way, the location of the imaged use-wear on the edge was visible in relation to the entire sample tool. The borders of the polish were marked using the polygon measurement function in ZENConnect to measure their 2D surface area. Only polish that was developed, bright or very bright, and continuous was selected for each polygon. A CSV table containing the surface area measurements was generated (Supplementary Material S26). The ZENConnect projects (including the images) are available on Zenodo (see Data availability).

3D models were processed using the GOM Inspect software both for macro use-wear documentation in CloudCompare and trajectory simulation in OFA. Since each software required a different alignment of the 3D models of each sample set, the meshes were processed twice. For documentation of edge damage using CloudCompare, the pre- and post-experiment 3D models were aligned to each other using the set matrix and align functions in GOM Inspect. Then the meshes were cleaned and any holes were filled. Excess data was removed by cropping the mesh.

For trajectory simulation in OFA, the 3D model of the relative position scan was orientated using the set matrix function. The cutting groove was orientated along the x-axis, with the start of the cutting groove at the origin of the 3D coordinate system. The edge of the contact material was orientated along the y-axis. Both the 3D model of the tool sample and the contact material were then aligned on the relative position scan using the pre-alignment and local best-fit functions (sample) and the 3-point alignment function (contact material). Note that for FLT8-1/BP-1, no relative position scan was acquired^37^, so a right angle between the 3D models of the sample and the bone plate was adjusted in GOM Inspect using the set matrix function. Each 3D model was then cleaned, closed, and cropped following the same steps as listed above. Lastly, the 3D model of the sample was translated negatively on the z-axis to create contact between the 3D models of the tool and its contact material. In this way, the position of the sample set at the starting point of the last experimental stroke was recreated digitally.

### Macroscopic damage visualisation

We consider macro use-wear damage as material loss to the edge of tools resulting from use. To visualise this wear on the edge, the 3D meshes of the pre-experimental (i.e., undamaged) and post-experimental (i.e., damaged) were compared using the cloud-to-mesh function in CloudCompare (v. 2.13.alpha) as demonstrated by Nora^42^. The C2M tool was used to compare surface deviations from mesh to mesh between each sample before (0strokes, reference mesh) and after (2000strokes, compared mesh) experimentation. The data produced from this gives a comparison of the meshes and therefore a visualisation of reduction of material on the edge (in this case edge damage) (see Nora^42^ and Nora et al. in preparation for further details).

Meshes were aligned by selecting and activating both the match bounding-box centres and fine registration (ICP) functions. Next, the meshes were compared using the cloud/mesh distance function. The properties of the “registered” mesh were then changed by making the colour scale visible. Comparison data was collected by selecting the “registered” mesh and exporting the histogram.

### OFA

The experimental trajectory was simulated in OFA (v2.1; available for download here: https://www.paleontology.uni-bonn.de/en/research/former-research-units/for-771-ofa/occlusal-fingerprint-analyser-ofa?set_language=en) to calculate contact areas for comparison with observed use-wear areas. The contact material 3D model remained static while the corresponding tool sample model was directed with scene-path points through the cutting groove. The trajectory of the tool simulated the last programmed (2000th) unilinear cutting movement of experimentation.

The OFA software takes advantage of 3D models and collision algorithms, and simulates antagonistic objects (teeth or tool/contact material) interactions digitally. When collisions between the 3D models occur (0.2 mm or closer), all vertices of the models involved are recorded. Then, the software takes this raw data, merges neighbouring vertices, and creates collision groups. These groups are displayed in the *collision viewer* that includes information such as size, the model(s) involved and, if applicable, the preceding collision of the group. The results in the *collision viewer* can be exported as a CSV file.

The 3D model of a sample and the corresponding contact material were imported into the software in place from STL files. A minimum of three scene-path points was defined to create a trajectory. The points were set by selecting the 3D model of the tool and the add scene-path point function, which added a triangle at the tool’s centre of gravity. From here, path points were placed to generate the trajectory using the translation function. The first point was always translated positively on the z-axis and negatively on the y-axis. The negative y translation was necessary to prevent the sample from getting stuck at the start of the cutting groove. The second point always corresponded to the tool's centre of gravity at the start of the cutting groove. No translation was required for this point. The last point was placed at + 140 mm on the x-axis at the end of the cutting groove. For the case of FLT8-1, this point was set at + 170 mm. Depending on the topography of the tool and contact material surface, it was sometimes necessary to set further points to guide the sample to move in the cut groove.

Additional settings were adjusted and kept identical for all simulations. The maximum degree of break-free was set to 100. Although arbitrary for this application, during some trial simulations, when the degree of break-free was set to lower (20) or higher (350) values, the tool diverted from the cutting groove and the trajectory aborted. The default setting for the set distance of 0.2 mm was accepted, meaning that contact was recorded anytime the 3D models came within 0.2 mm distance. A step distance of 0.5 was chosen for the final simulations. The lower the step-distance value, the higher the number of steps added to the trajectory and the more fine-grained the calculated contact areas.

After each collision trajectory, data on contact areas was collected by exporting CSV tables of the collision groups and bar charts using the diagram function. The *show collision distance* function was activated which shows the distance between the interacting models. It spans a gradient from blue (far) to red (close). Images of the contact areas on the tool were acquired using the *grab image* function. The OFA projects (with 3D models) are available on Zenodo (see Data availability).

### Statistical analysis

Sensor data recorded throughout the experimental cutting strokes with the SMARTTESTER was analysed in R. The analysis included descriptive statistics and plotting the variables velocity, force, penetration depth, and friction. Velocity and force were examined to check the degree of control of the experiments. Penetration depth was relevant to study tool efficacy, and friction was looked at to understand the wear mechanics. All packages used are cited in the R research compendium (see Data availability).

### Supplementary Information

Supplementary Information 1.

Supplementary Information 2.

Supplementary Information 3.

## Data Availability

Supplementary material is available in open-access at: Experimental protocol: 10.17504/protocols.io.261ge346dl47/v1. OFA projects, 3D and use-wear data: 10.5281/zenodo.11385991, 10.5281/zenodo.10429335. GitHub repository with statistical analysis: https://github.com/haennah/OFA_lithics/tree/v1.0 and its release on Zenodo 10.5281/zenodo.8205688.
