# Supplementary material for: An experimental approach on dynamic occlusal fingerprint analysis to simulate use-wear localisation and development on stone tools
Source: Sci Rep. 2024 Aug 29;14:20084. doi: 10.1038/s41598-024-70265-1 (PMC11362603; doi:10.1038/s41598-024-70265-1)
Supplement: Supplementary file 1 — Supplementary Information 1. [file 41598_2024_70265_MOESM1_ESM.pdf]

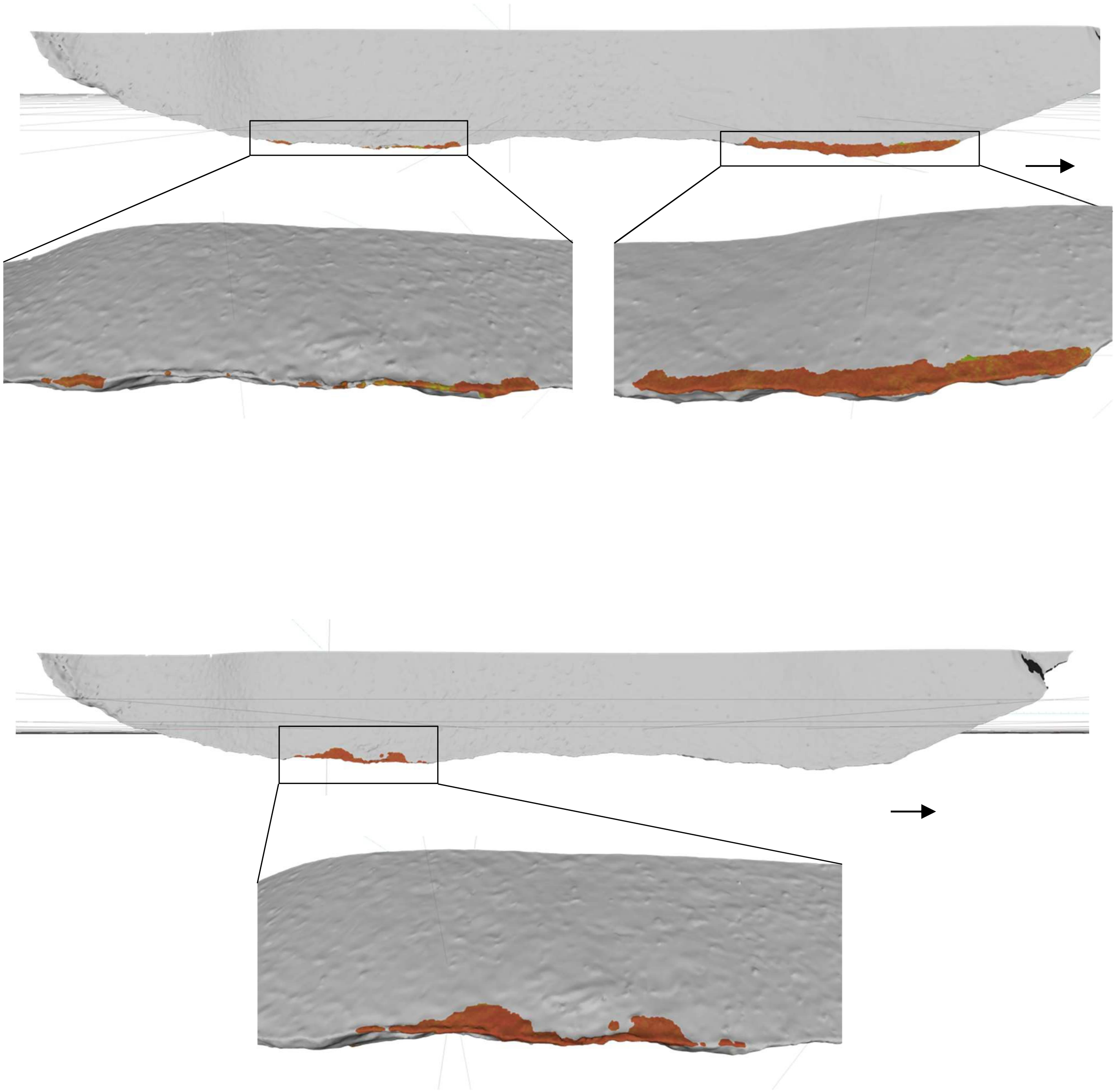

Top: Contact areas on the ventral side of tool sample FLT13-12 at distance 13 mm (step 29) on the set trajectory.

Bottom: Contact areas on the ventral side of tool sample FLT13-12 at distance 28 mm (step 63). Contact areas are shown as a gradient of the collision distance between both 3D models from red to blue: red illustrates a close collision. The arrow indicates the direction of the stroke. The length of the tool is 71.9 mm.

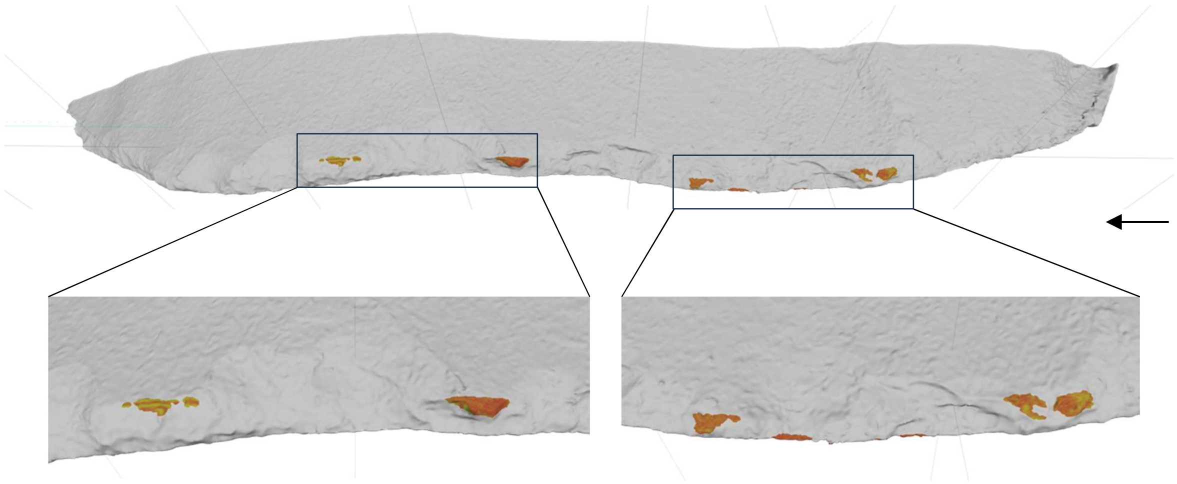

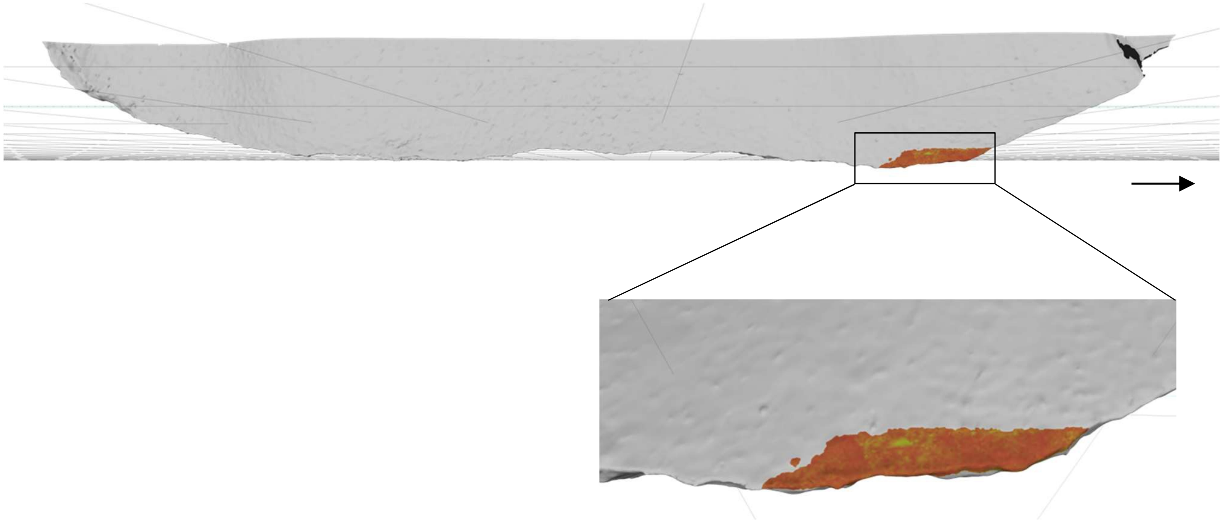

Top: Contact areas on the dorsal side of tool sample FLT13-12 at distance 29 mm (step 64) on the set trajectory.

Bottom: Contact areas on the ventral side of tool sample FLT13-12 at distance 97 mm (step 214) on the set trajectory. Contact areas are shown as a gradient of the collision distance between both 3D models from red to blue: red illustrates a close collision. The arrow indicates the direction of the stroke. The length of the tool is 71.9 mm.

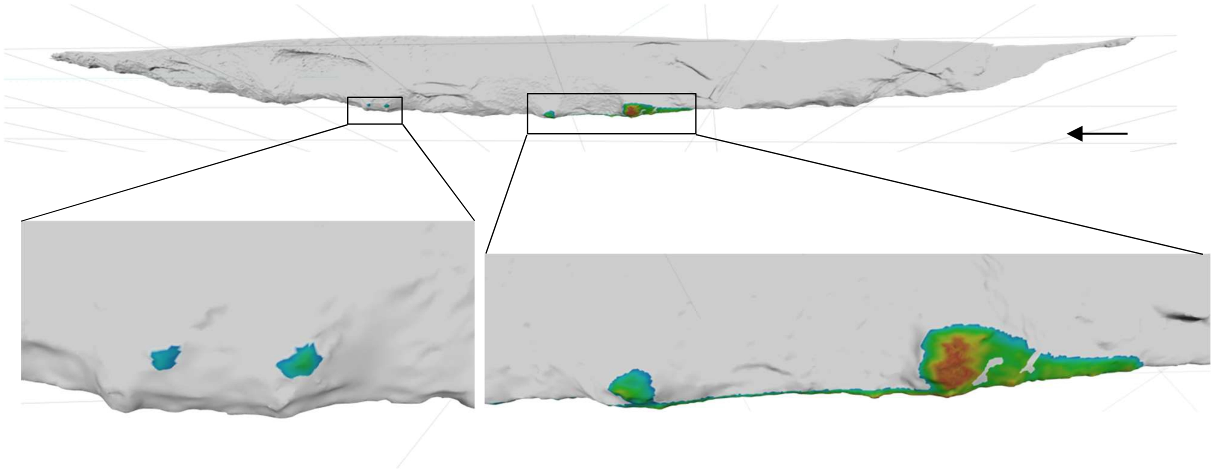

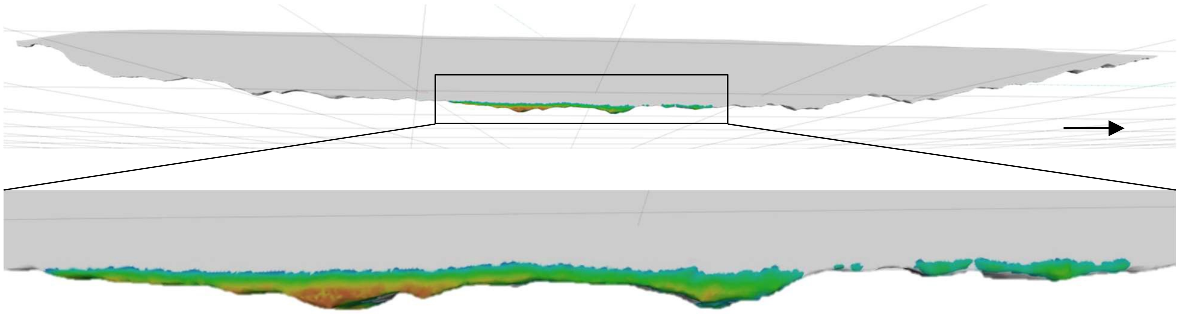

Top: Contact areas on the dorsal side of tool sample FLT13-1 at distance 18 mm (step 39) on the set trajectory.

Bottom: Contact areas on the ventral edge of tool sample FLT13-1 at distance 18 mm. Contact areas are shown as a gradient of the collision distance between both 3D models from red to blue: red illustrates a close collision and blue is a less close collision. The arrow indicates the direction of the stroke. The length of the tool is 83.4 mm.

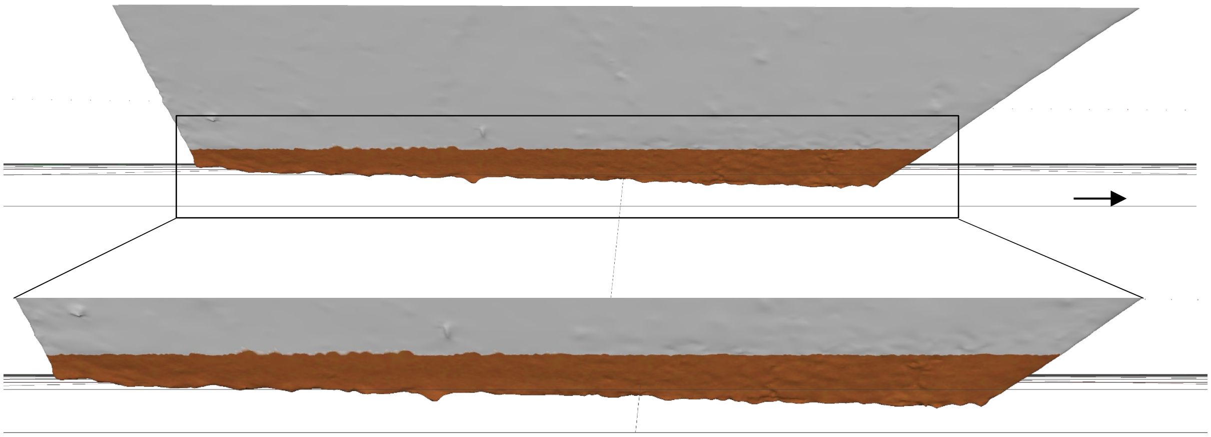

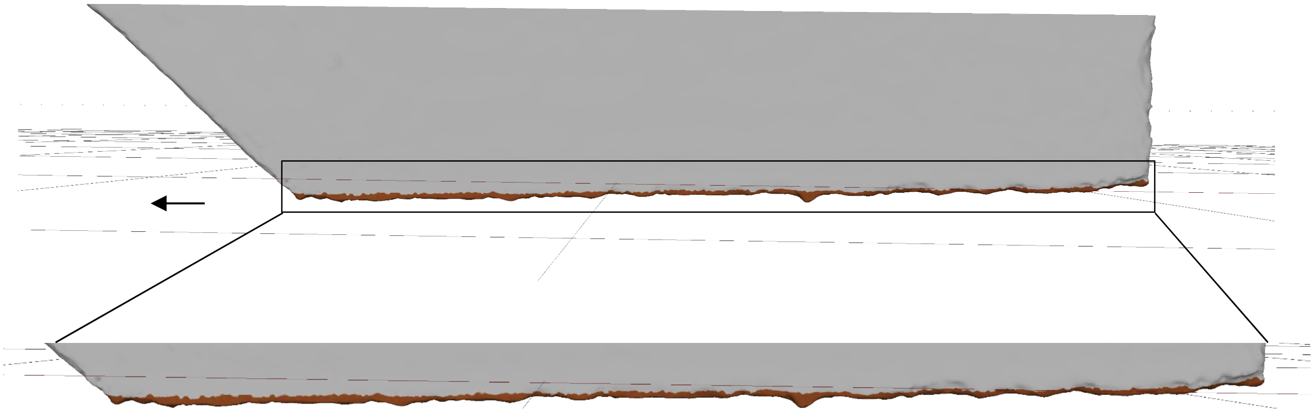

Top: Contact areas on the dorsal side of standard cut tool sample FLT8-13 at distance 51 mm (step 120) on the set trajectory.

Bottom: Contact areas on the ventral side of standard cut tool sample FLT8-13 at distance 51 mm (step 120). Contact areas are shown as a gradient of the collision distance between both 3D models from red to blue: red illustrates a close collision. The arrow indicates the direction of the stroke. The length of the tool is 24.4 mm.

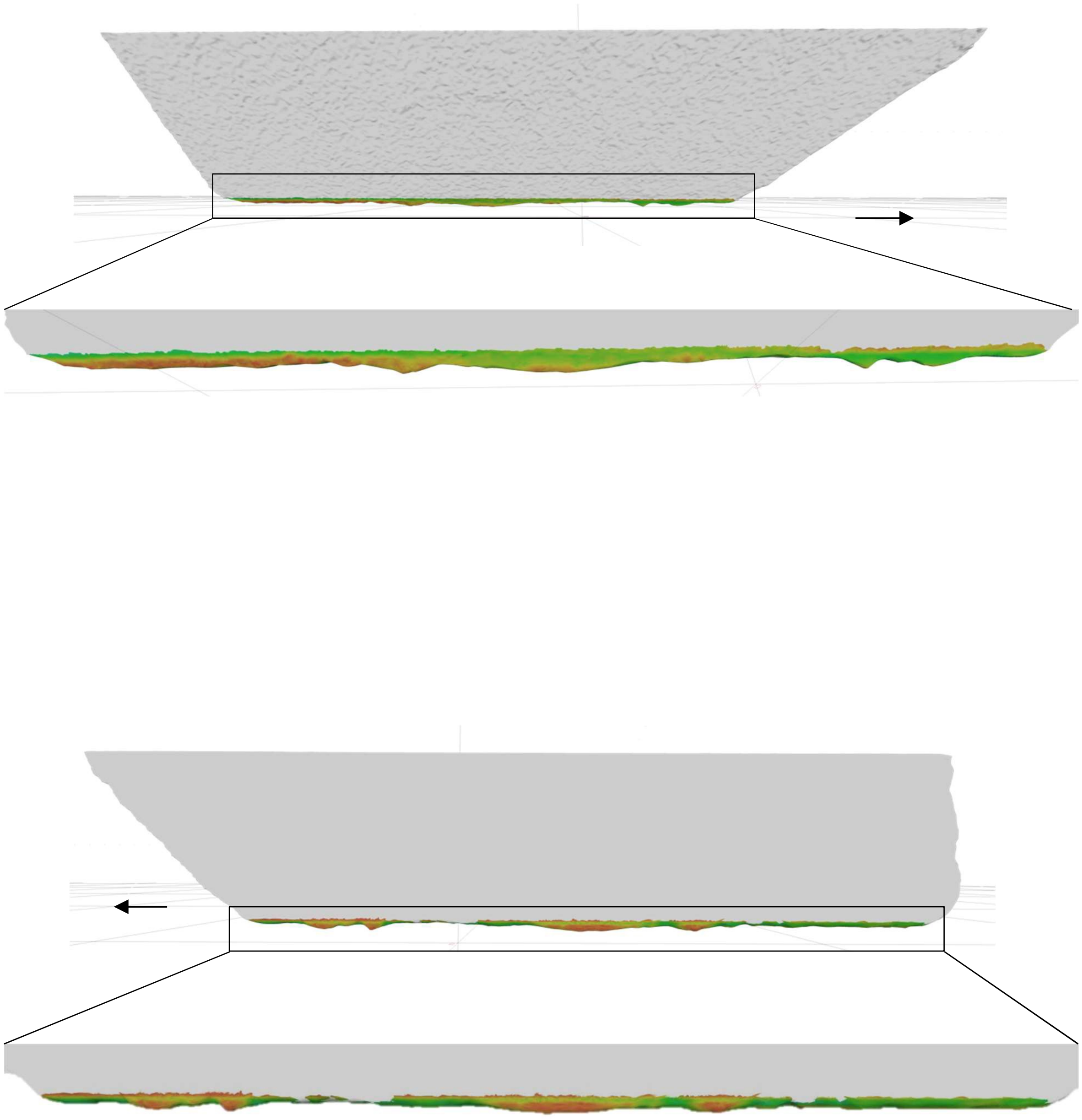

Top: Contact areas on the dorsal side of standard cut tool sample FLT8-1 at distance 12 mm (step 28) on the set trajectory.

Bottom: Contact areas on the ventral side of standard cut tool sample FLT8-1 at distance 12 mm (step 28). Contact areas are shown as a gradient of the collision distance between both 3D models from red to blue: red illustrates a close collision. The arrow indicates the direction of the stroke. The length of the tool is 24.6 mm.

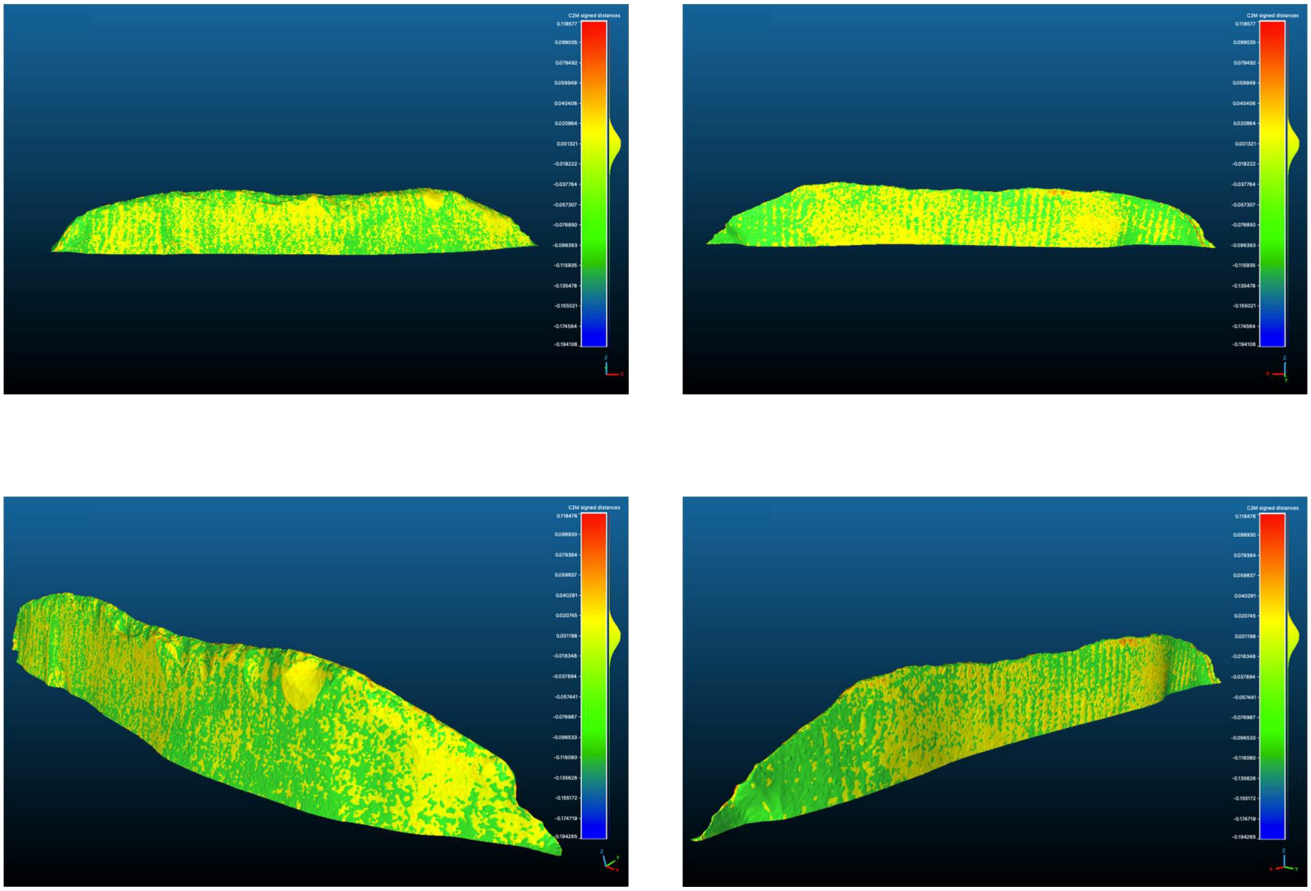

Cloud-to-mesh edge reduction comparison of tool sample FLT13-12 dorsal (left) and ventral (right) in CloudCompare (v. 2.13.alpha, 2023). The length of the tool is 71.9 mm.

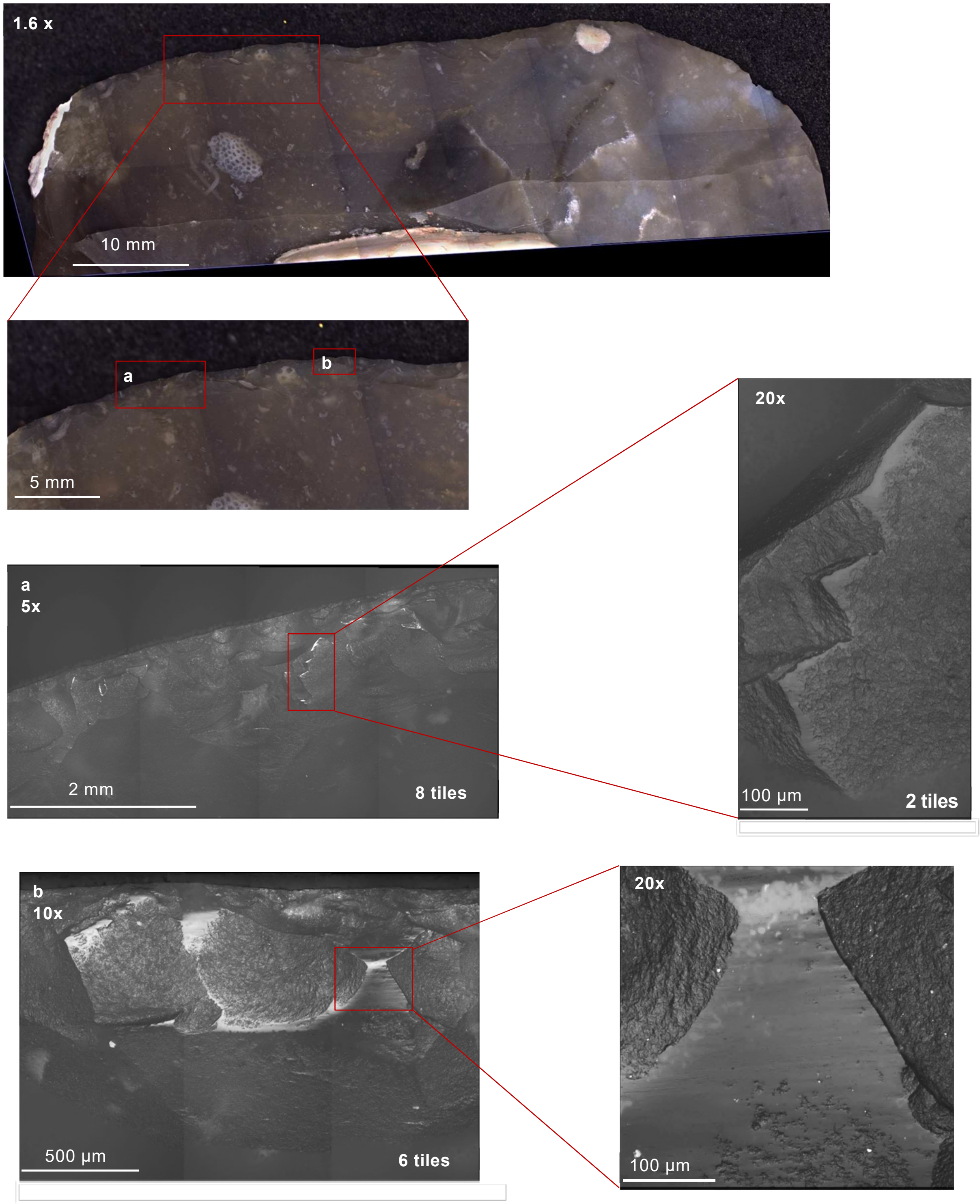

EDF-stitched images of polished areas on the dorsal side of tool sample FLT13-12.

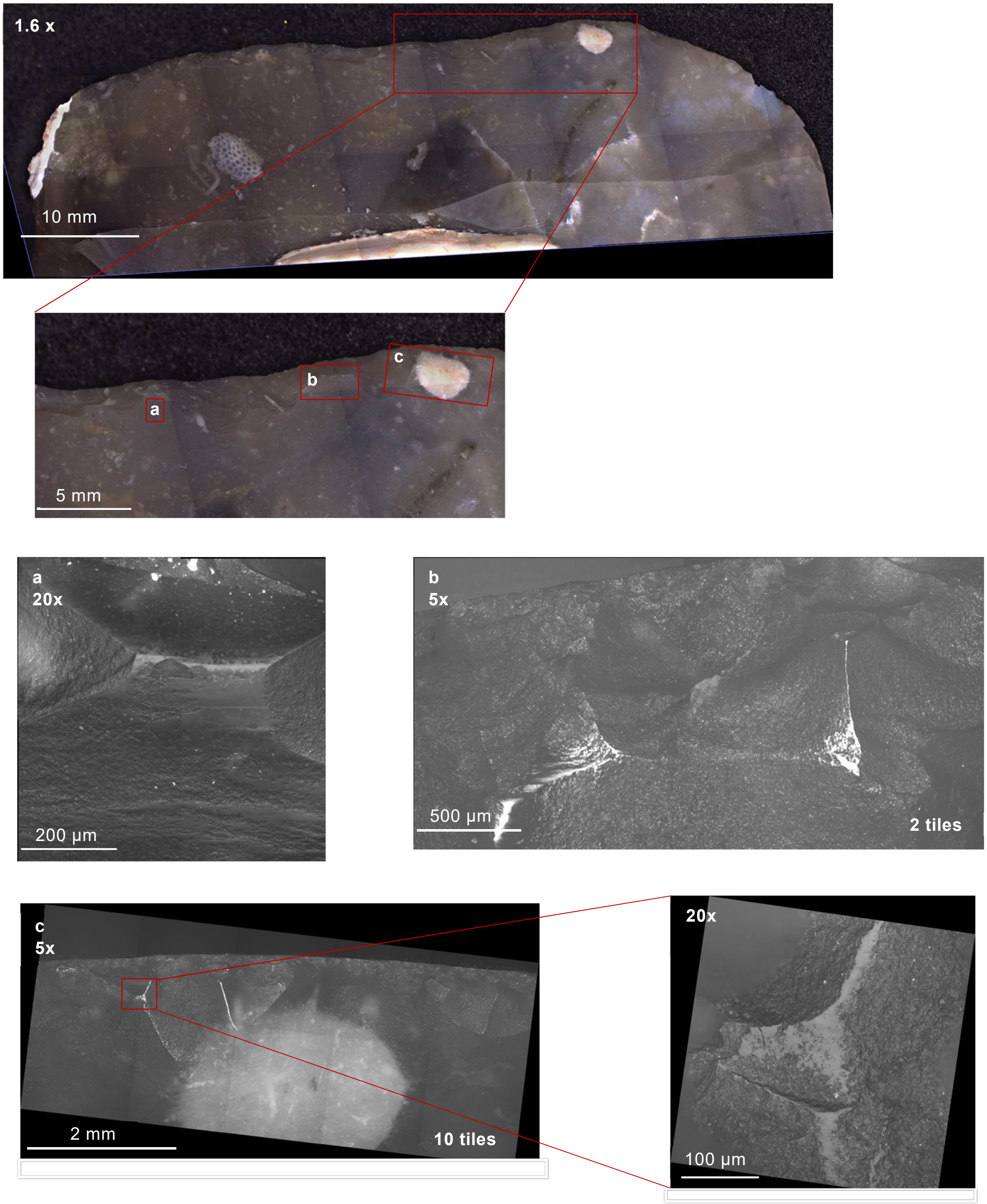

EDF-stitched images of polished areas on the dorsal side of tool sample FLT13-12.

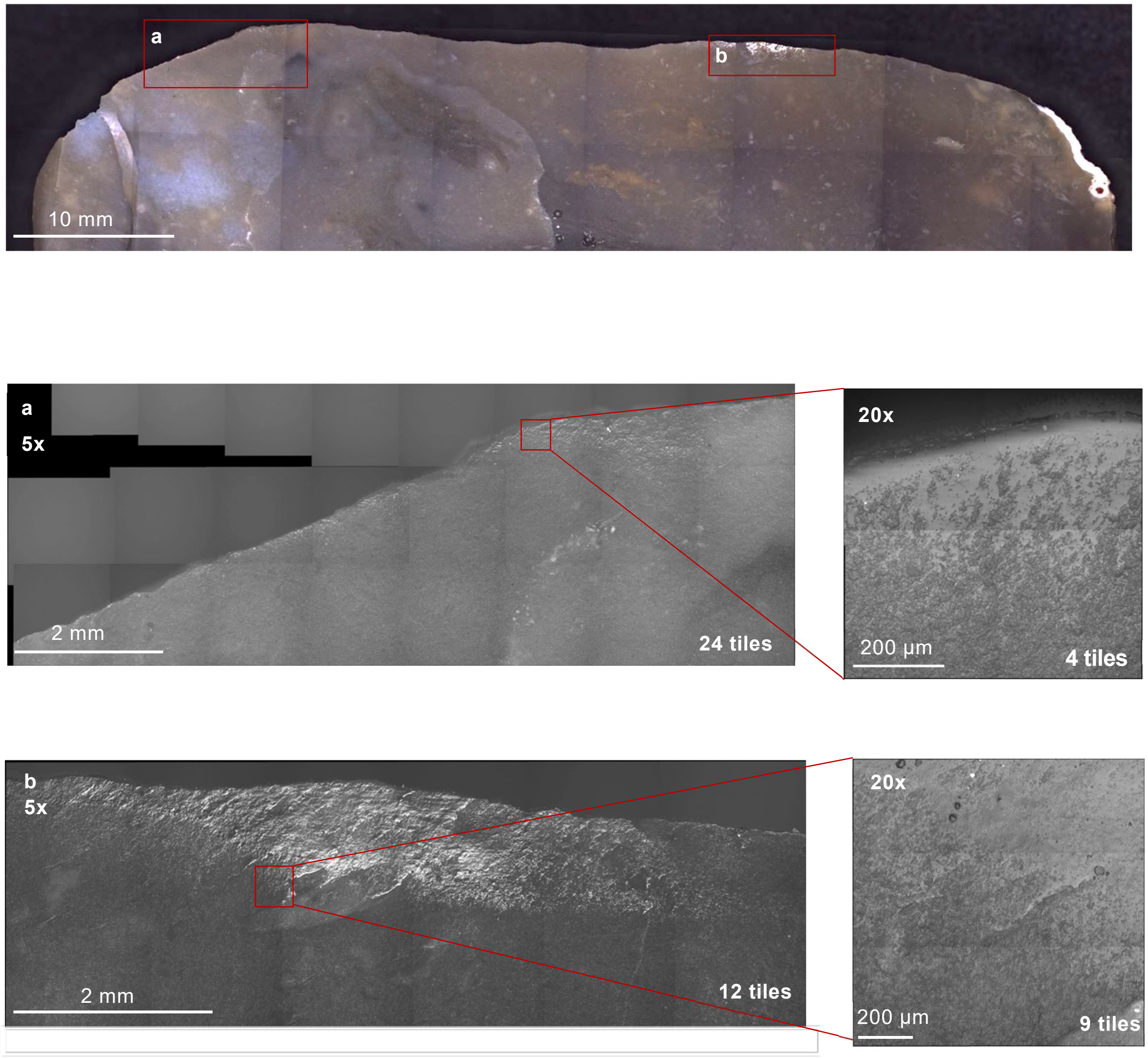

EDF-stitched images of polished areas on the ventral side of tool sample FLT13-12.

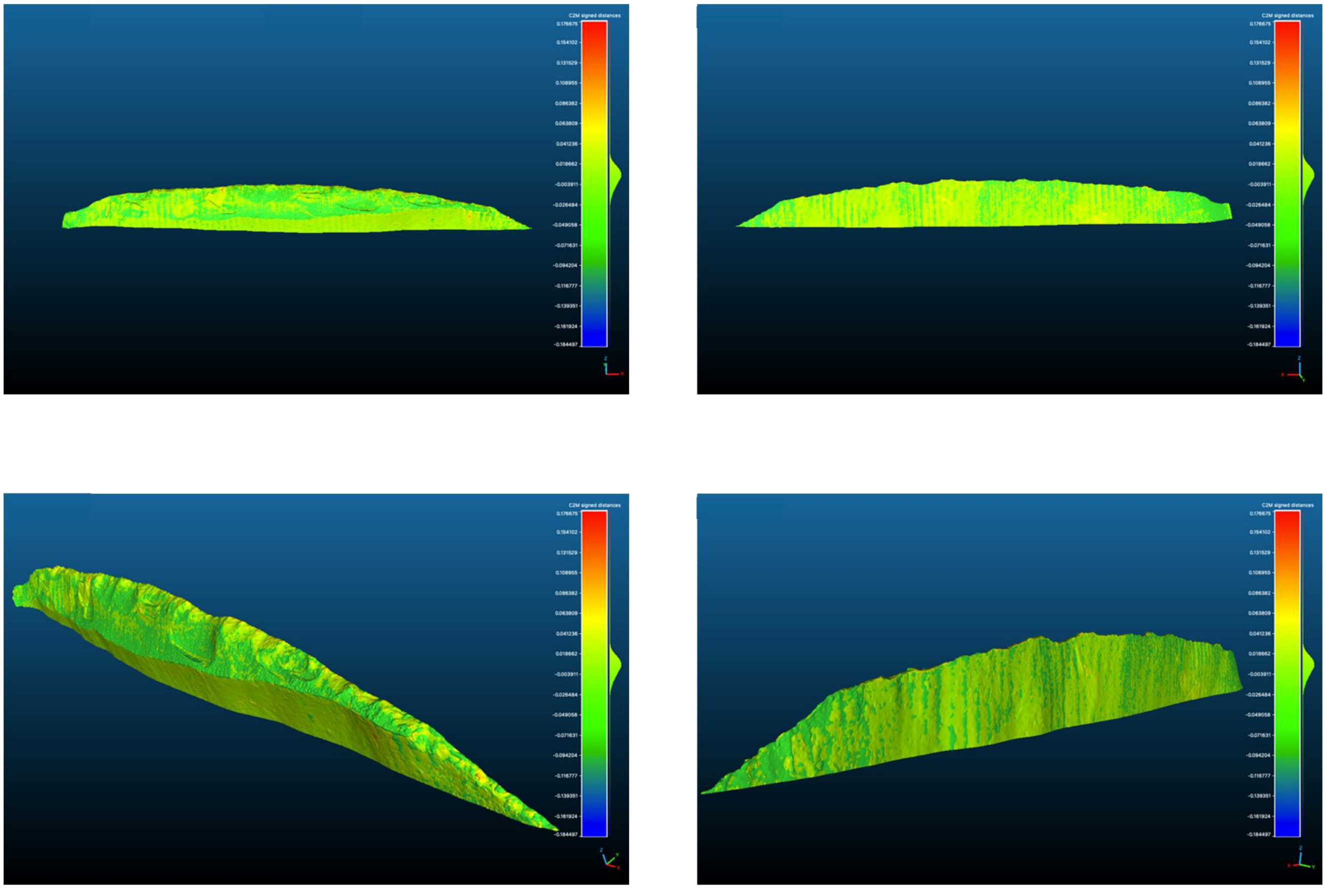

Cloud-to-mesh edge reduction comparison of tool sample FLT13-1 dorsal (left) and ventral (right) in CloudCompare (v. 2.13.alpha, 2023). The length of the tool is 83.4 mm.

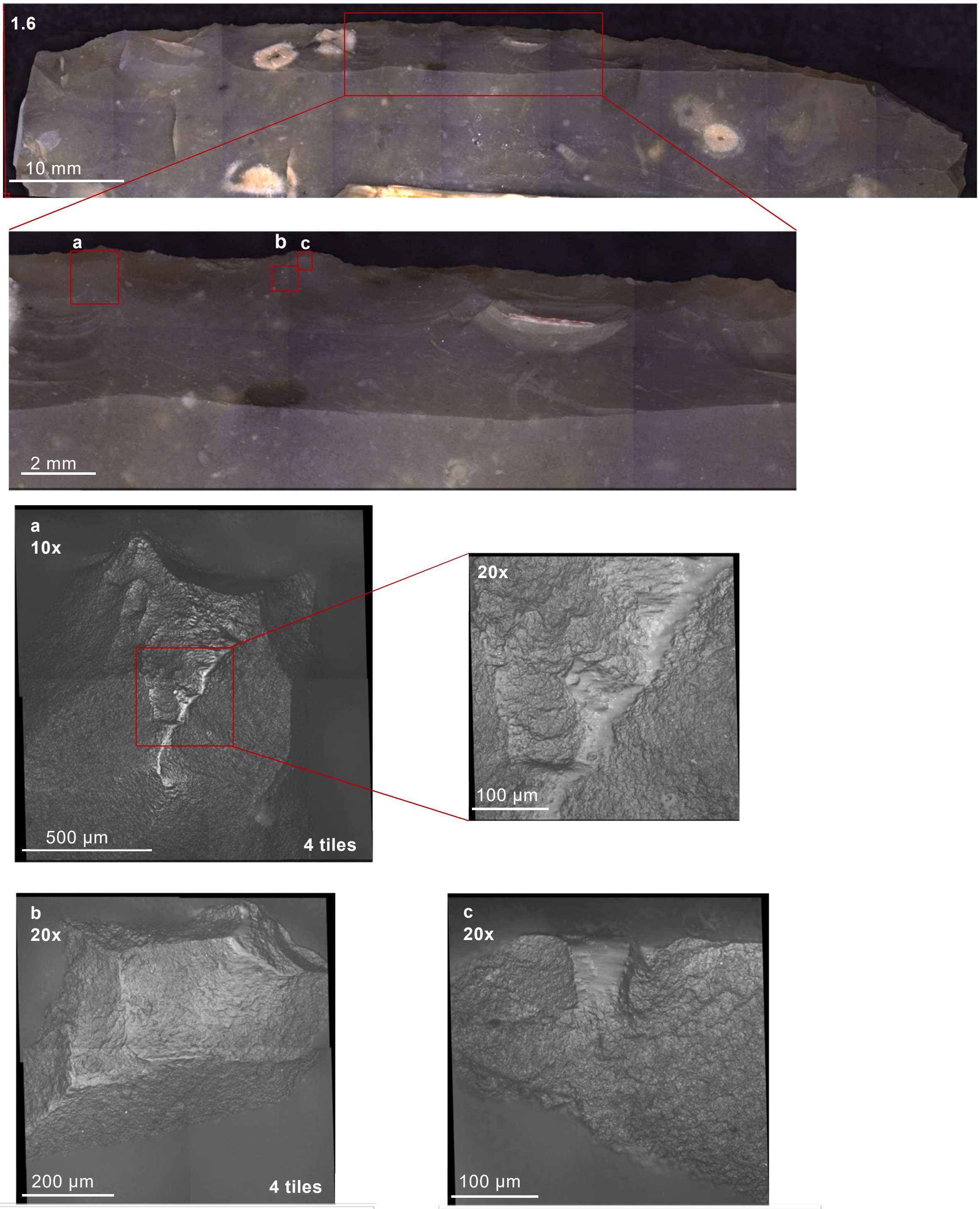

EDF-stitched images of polished areas on the dorsal side of tool sample FLT13-1.

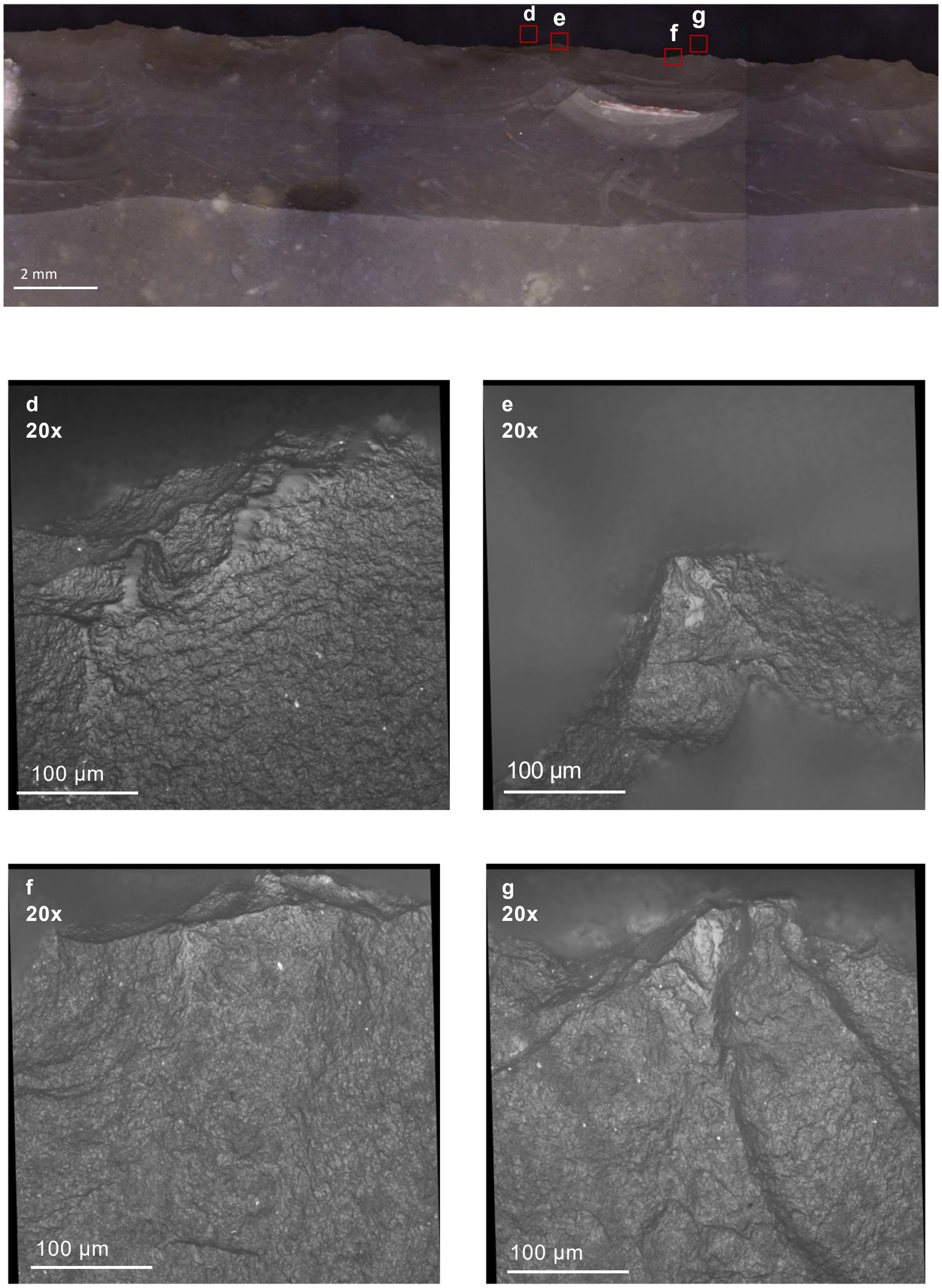

EDF-stitched images of polished areas on the dorsal side of tool sample FLT13-1.

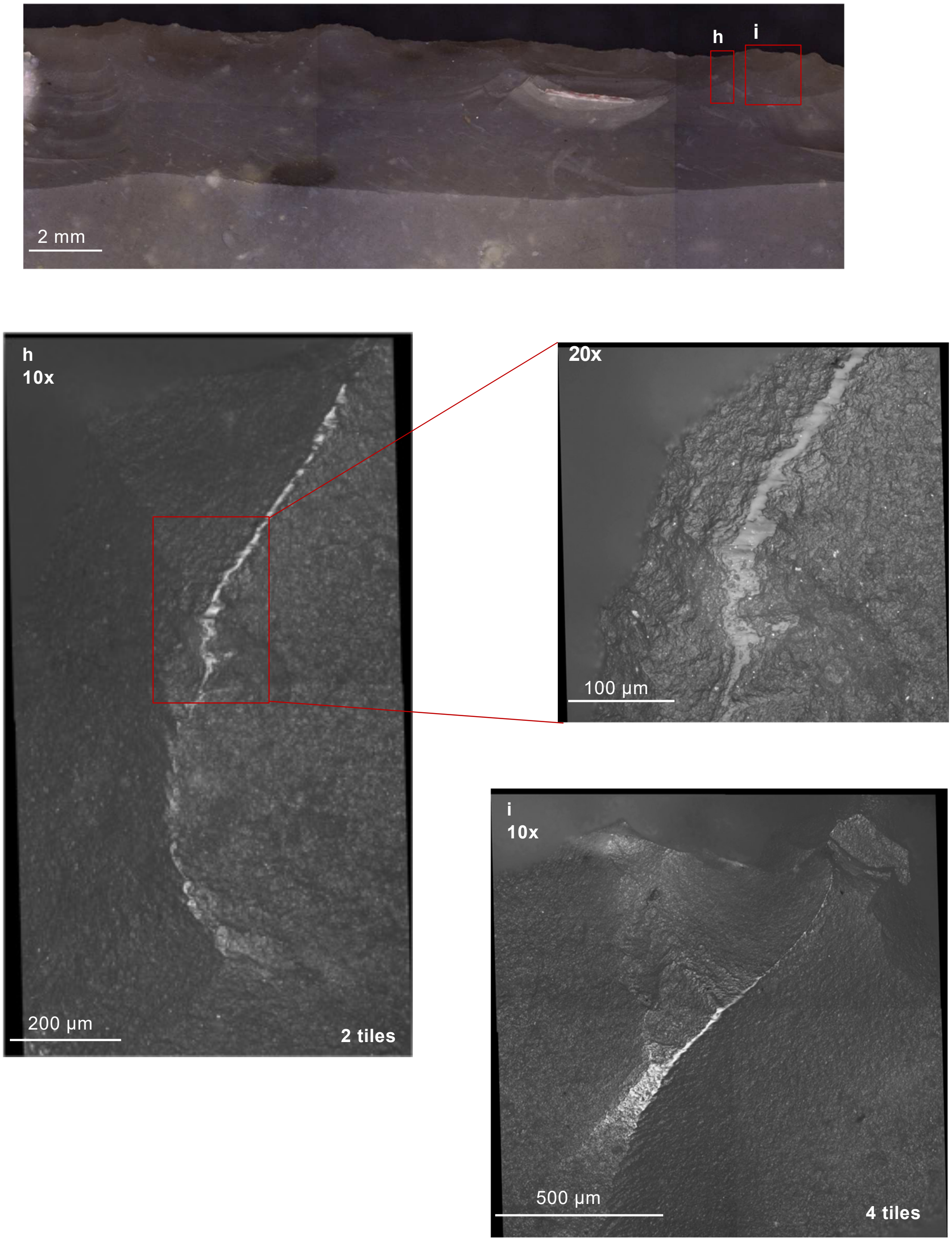

EDF-stitched images of polished areas on the dorsal side of tool sample FLT13-1.

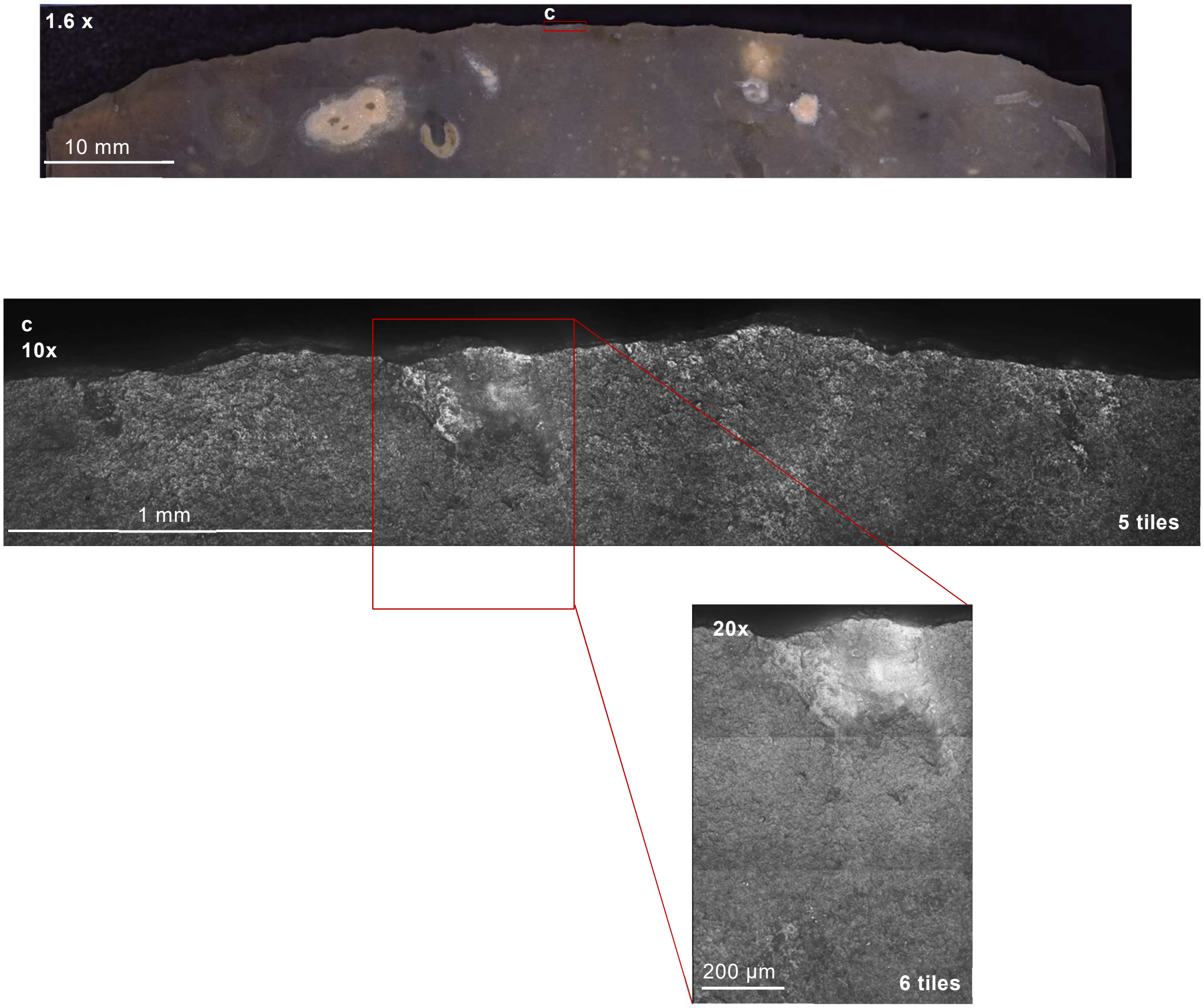

EDF-stitched images of polished areas on the ventral side of tool sample FLT13-1.

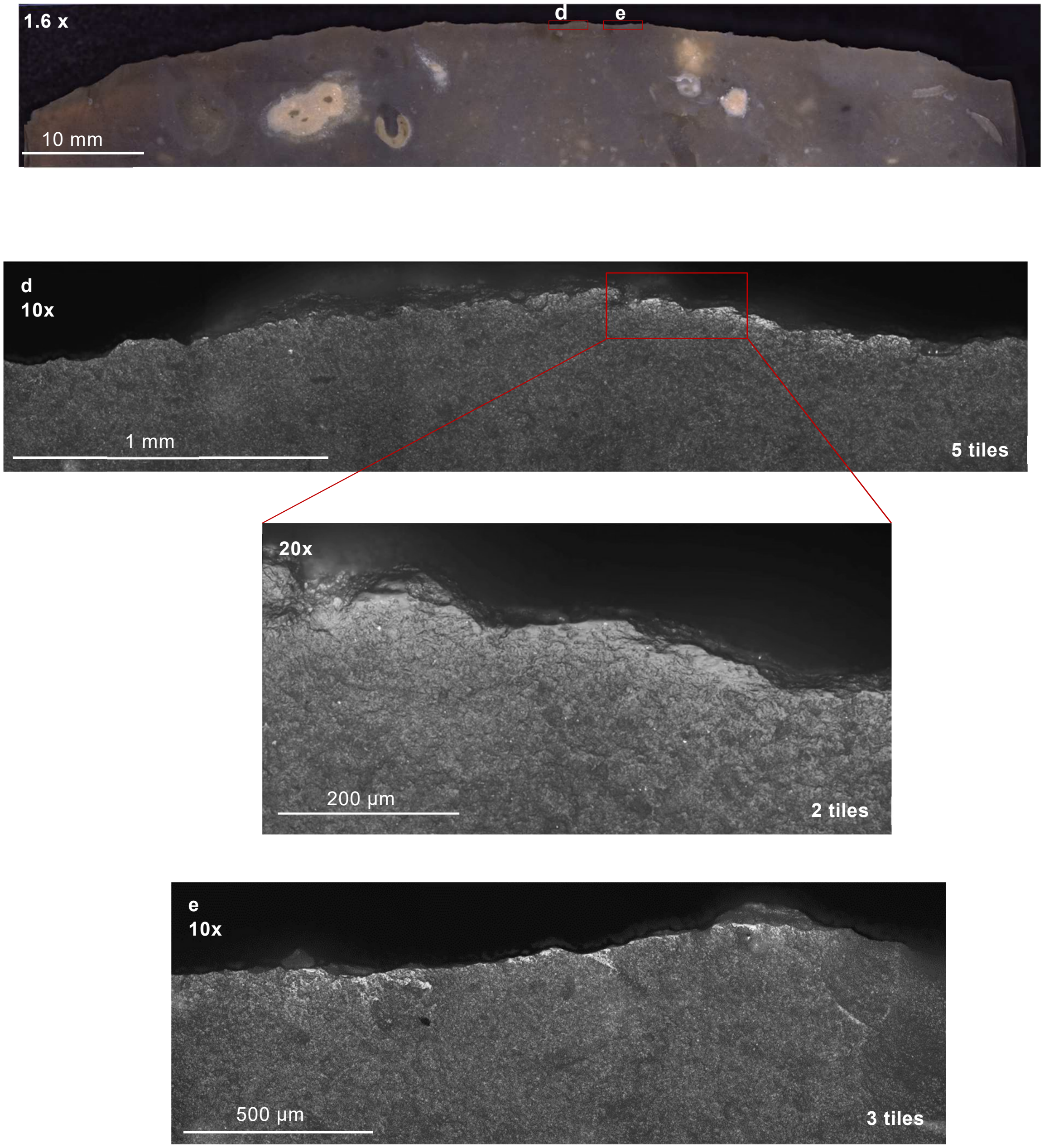

EDF-stitched images of polished areas on the ventral side of tool sample FLT13-1.

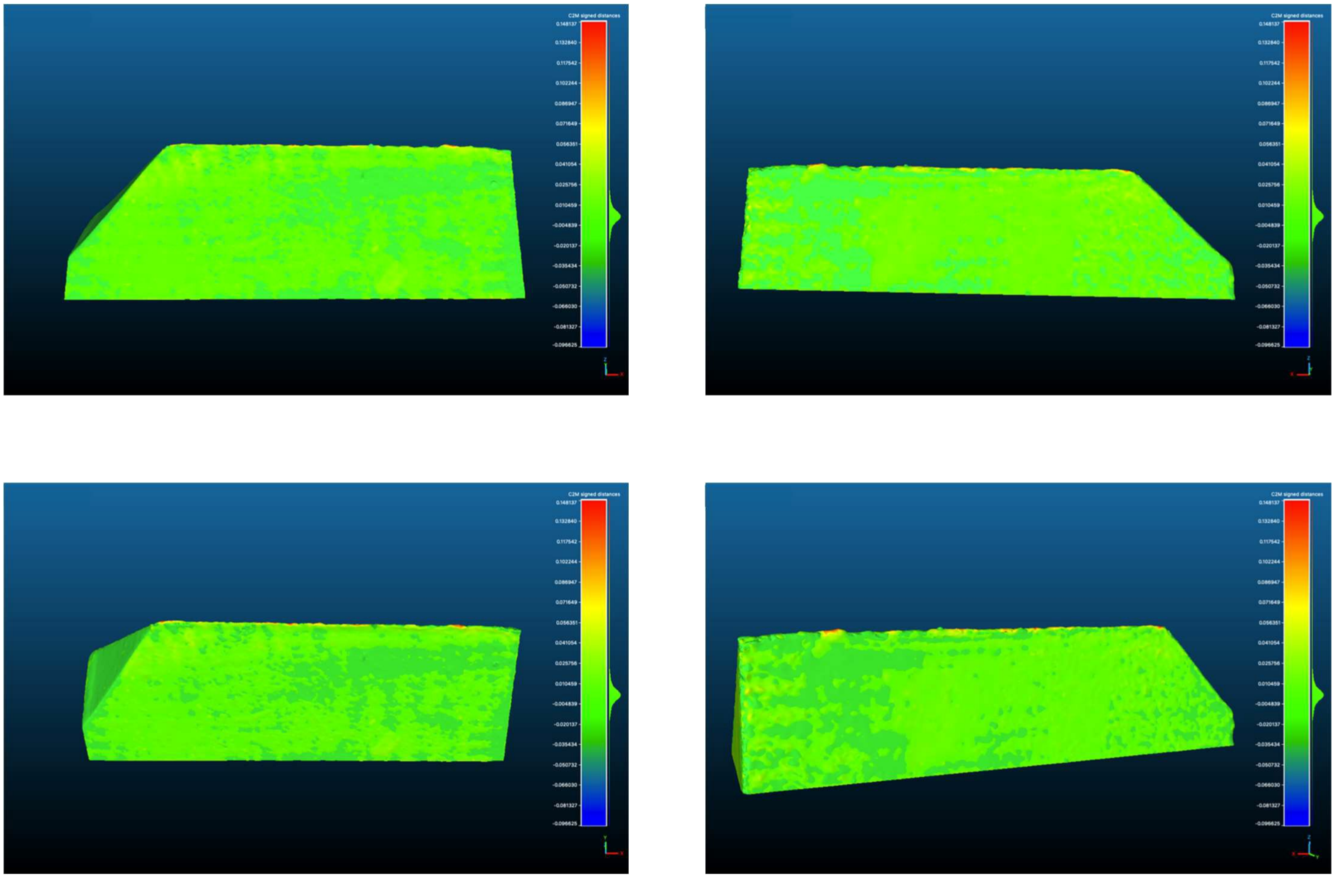

Cloud-to-mesh edge reduction comparison of standard cut tool sample FLT8-13 dorsal (left) and ventral (right) in CloudCompare (v. 2.13.alpha, 2023). The length of the tool is 24.4 mm.

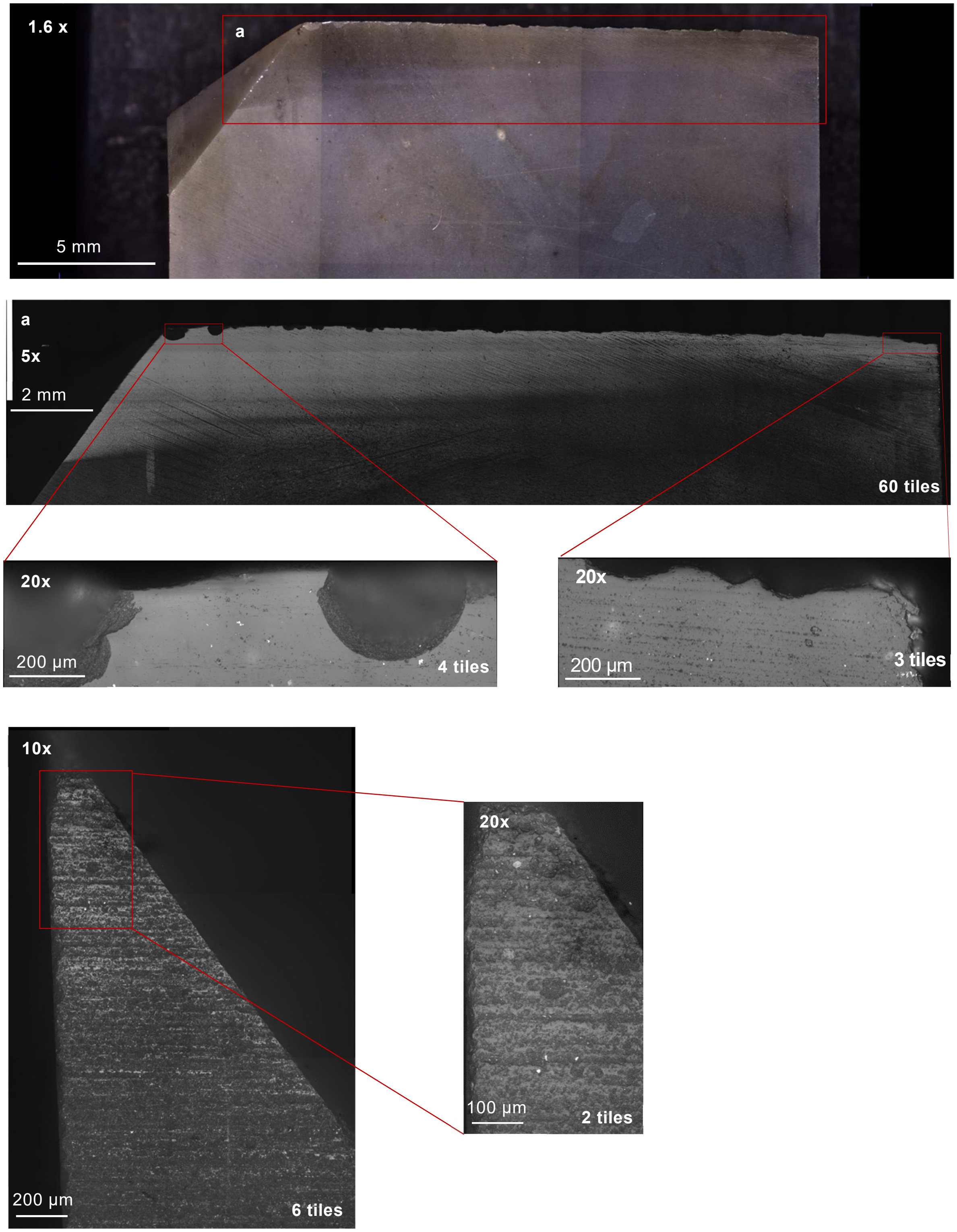

EDF-stitched images of polished areas on the dorsal side (top) and chamfered-edge (bottom) of standard cut tool sample FLT8-13.

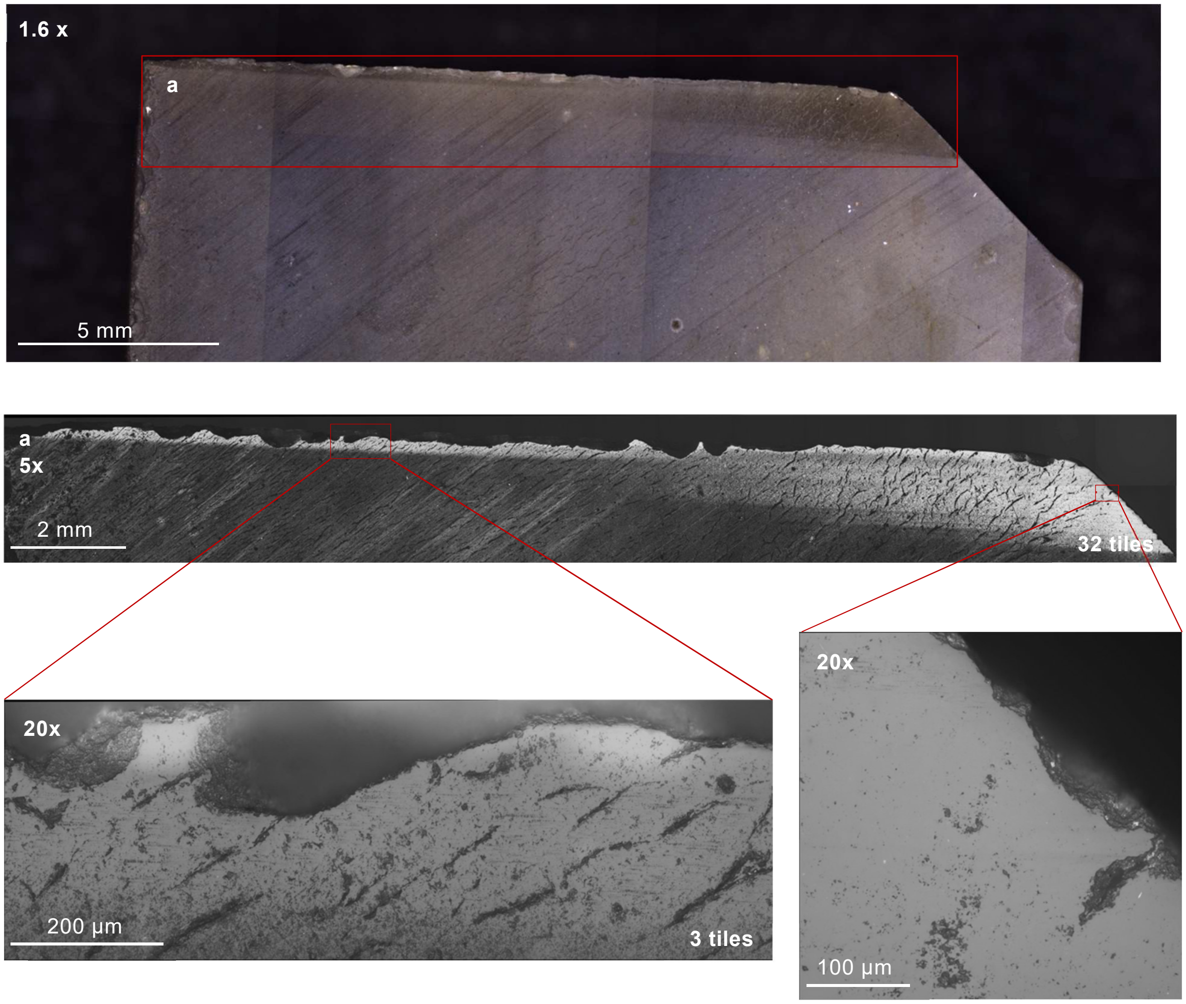

EDF-stitched images of polished areas on the ventral side of standard cut tool sample FLT8-13.

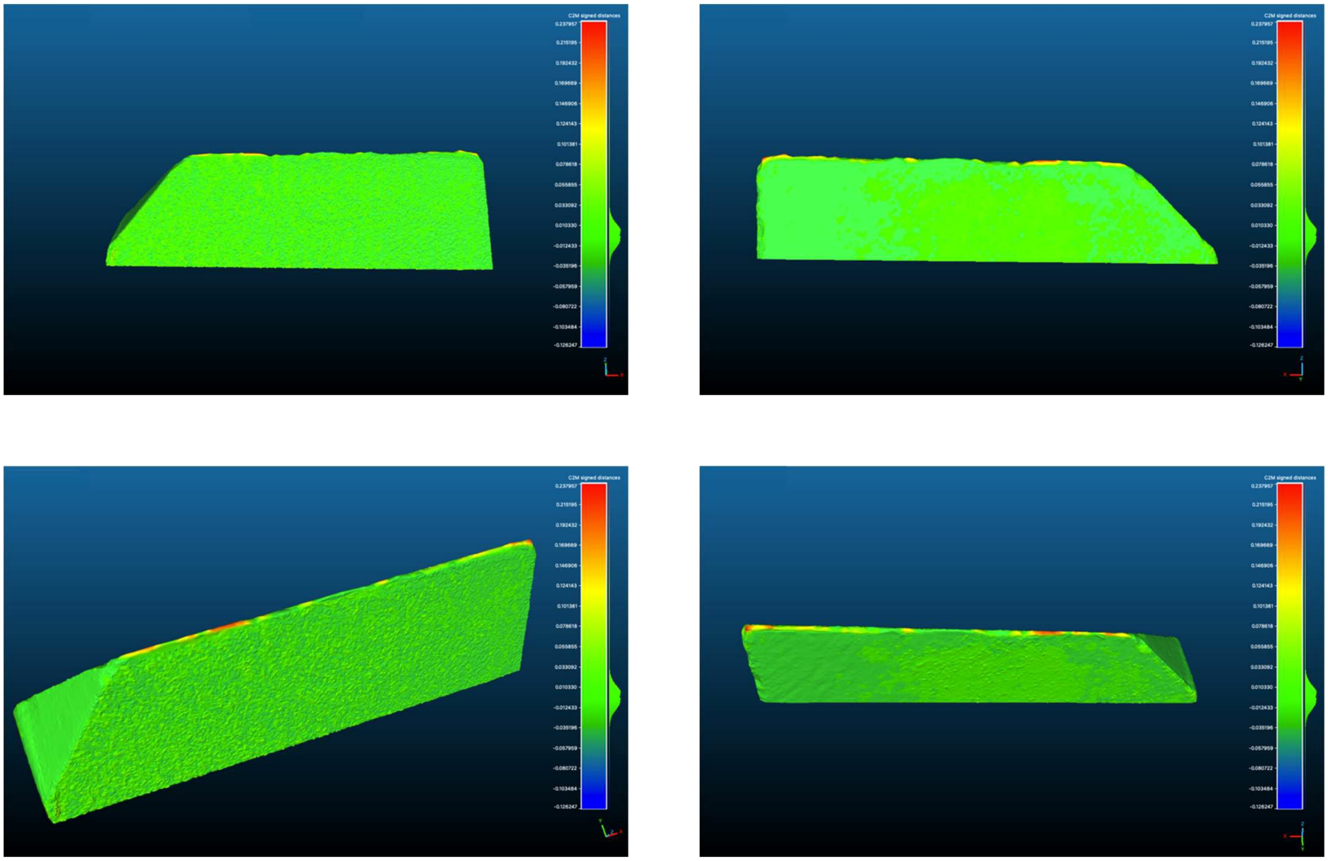

Cloud-to-mesh edge reduction comparison of standard cut tool sample FLT8-1 dorsal (left) and ventral (right) in CloudCompare (v. 2.13.alpha, 2023). The length of the tool is 24.6 mm.

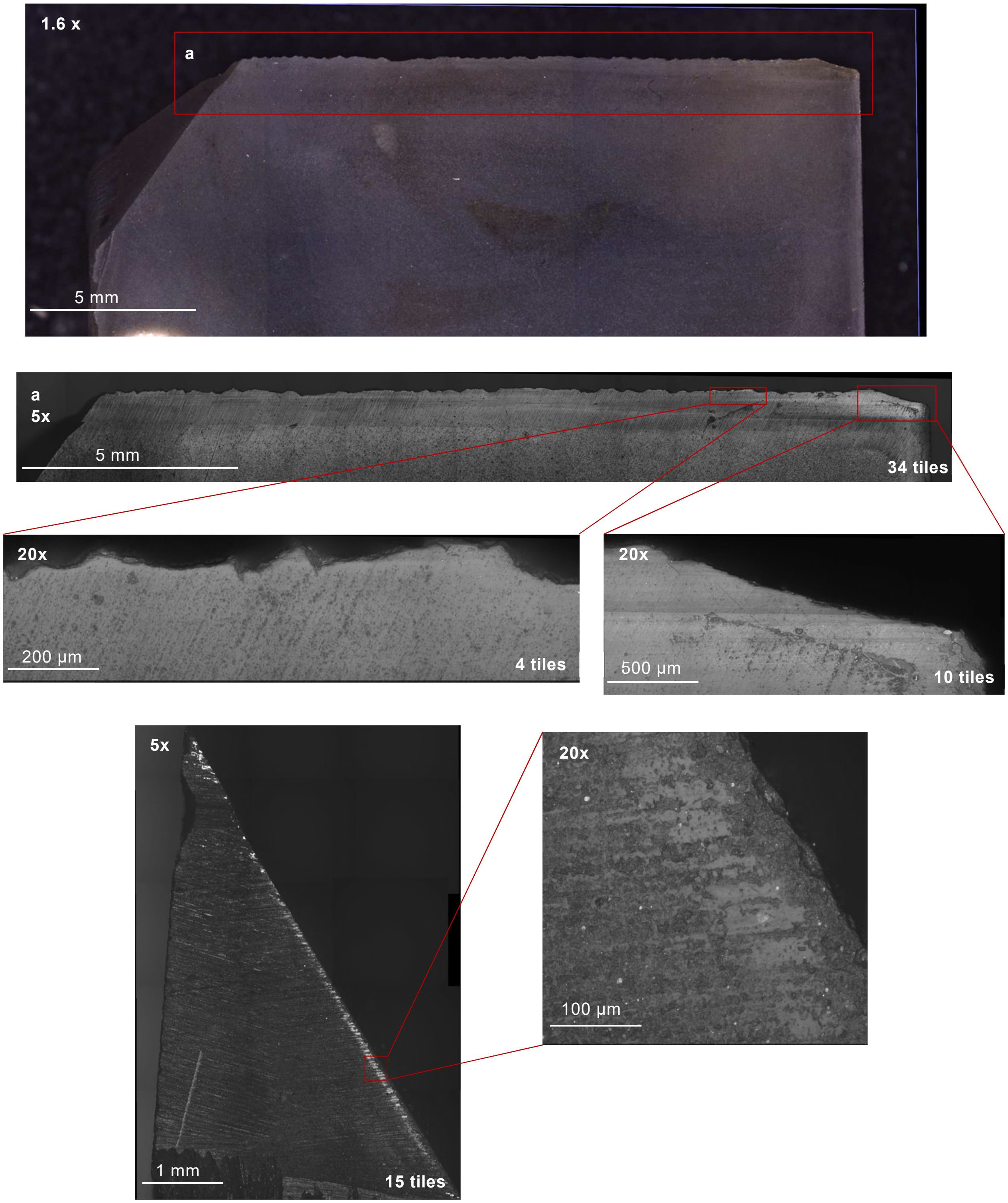

EDF-stitched images of polished areas on the dorsal side (top) and chamfered-edge (bottom) of standard cut tool sample FLT8-1.

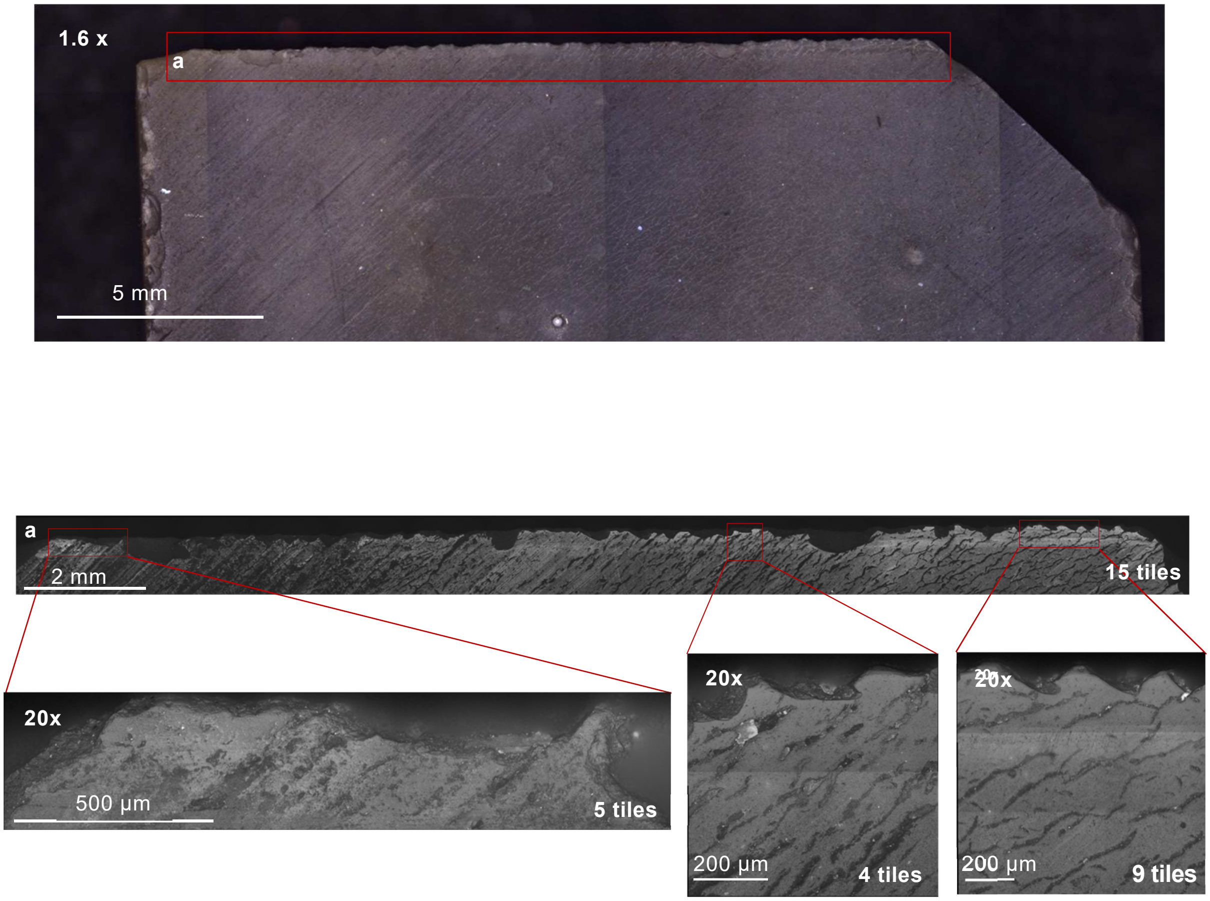

EDF-stitched images of polished areas on the ventral side of standard cut tool sample FLT8-1.

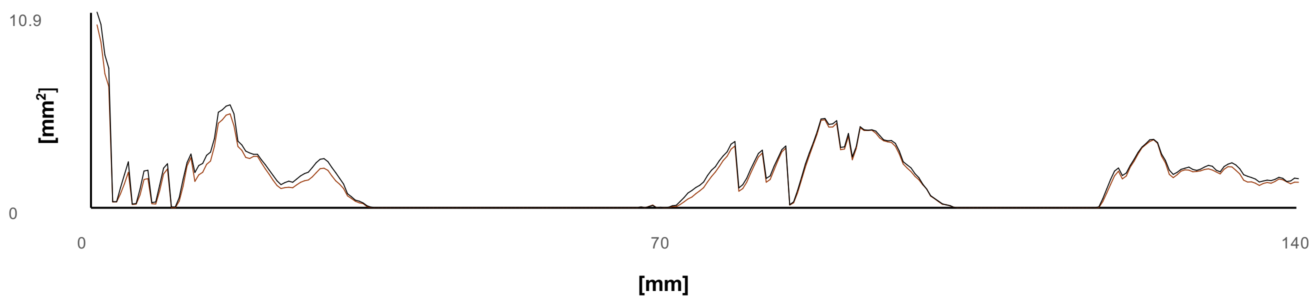

The surface area of contact calculated in OFA between knapped tool sample FLT13-12 (red) and wood plate OFA-WP1 (black) during a 140 mm trajectory.

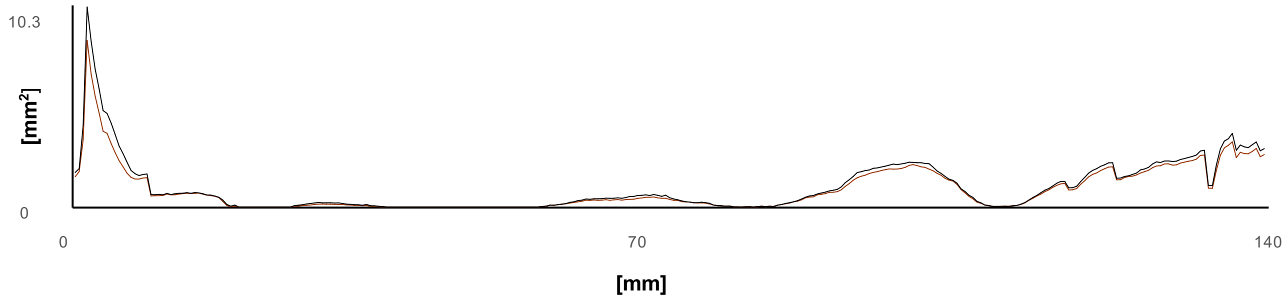

The surface area of contact calculated in OFA between knapped tool sample FLT13-1 (red) and bone plate OFA-BP1 (black) during the 140 mm trajectory.

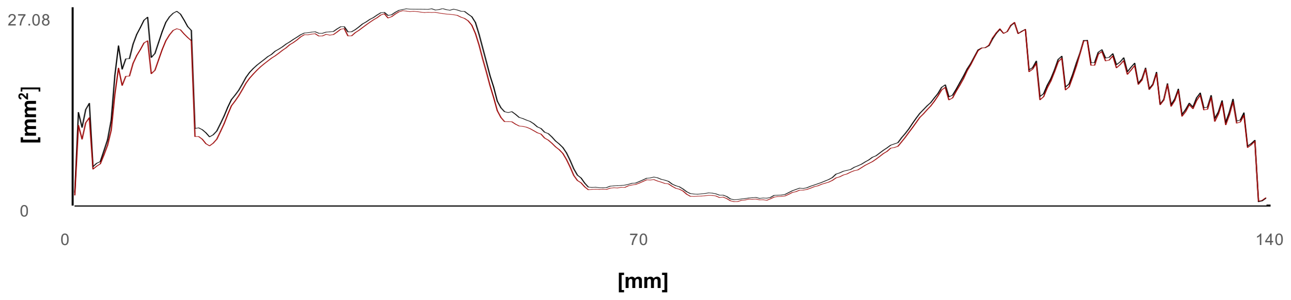

The surface area of contact calculated in OFA between standard cut tool sample FLT8-13 (red) and wood plate OFA-WP1 (grey) during the 140 mm trajectory.

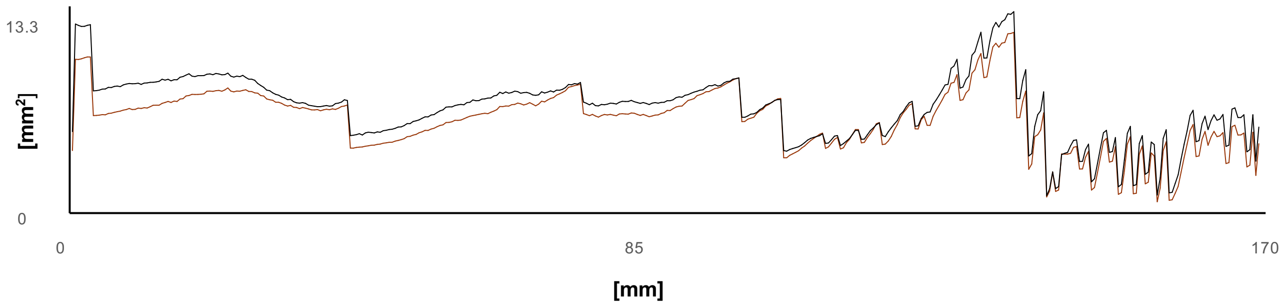

The surface area of contact calculated in OFA between standard cut tool sample FLT8-1 (red) and bone plate BP-cutting (grey) during the 170 mm trajectory.
