# Supplementary material for: An experimental approach on dynamic occlusal fingerprint analysis to simulate use-wear localisation and development on stone tools
Source: Sci Rep. 2024 Aug 29;14:20084. doi: 10.1038/s41598-024-70265-1 (PMC11362603; doi:10.1038/s41598-024-70265-1)
Supplement: Supplementary file 3 — Supplementary Information 3. [file 41598_2024_70265_MOESM3_ESM.pdf]

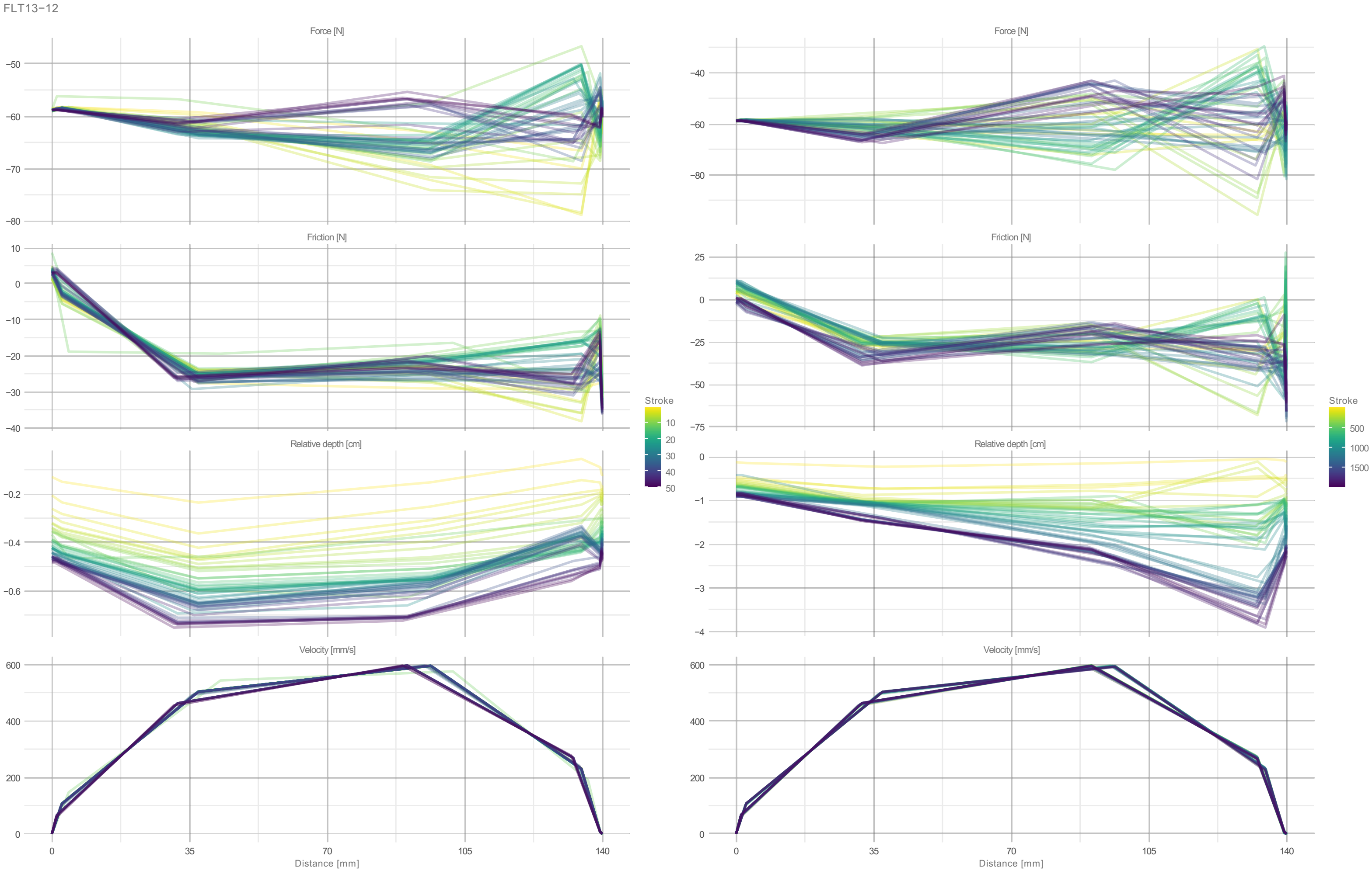

Sensor data (force, friction, relative depth and velocity) of unilinear strokes performed with knapped sample FLT13-12 on wood plate OFA-WP1. Sensor data is illustrated on the y-axis. Distance on the x-axis illustrates the position of the sample on the contact material from the origin of each stroke. The left block of graphs shows the first 50 strokes of the experiment. The right block of graphs shows all 2000 strokes. Lines illustrate every 40th cutting stroke from yellow to purple.

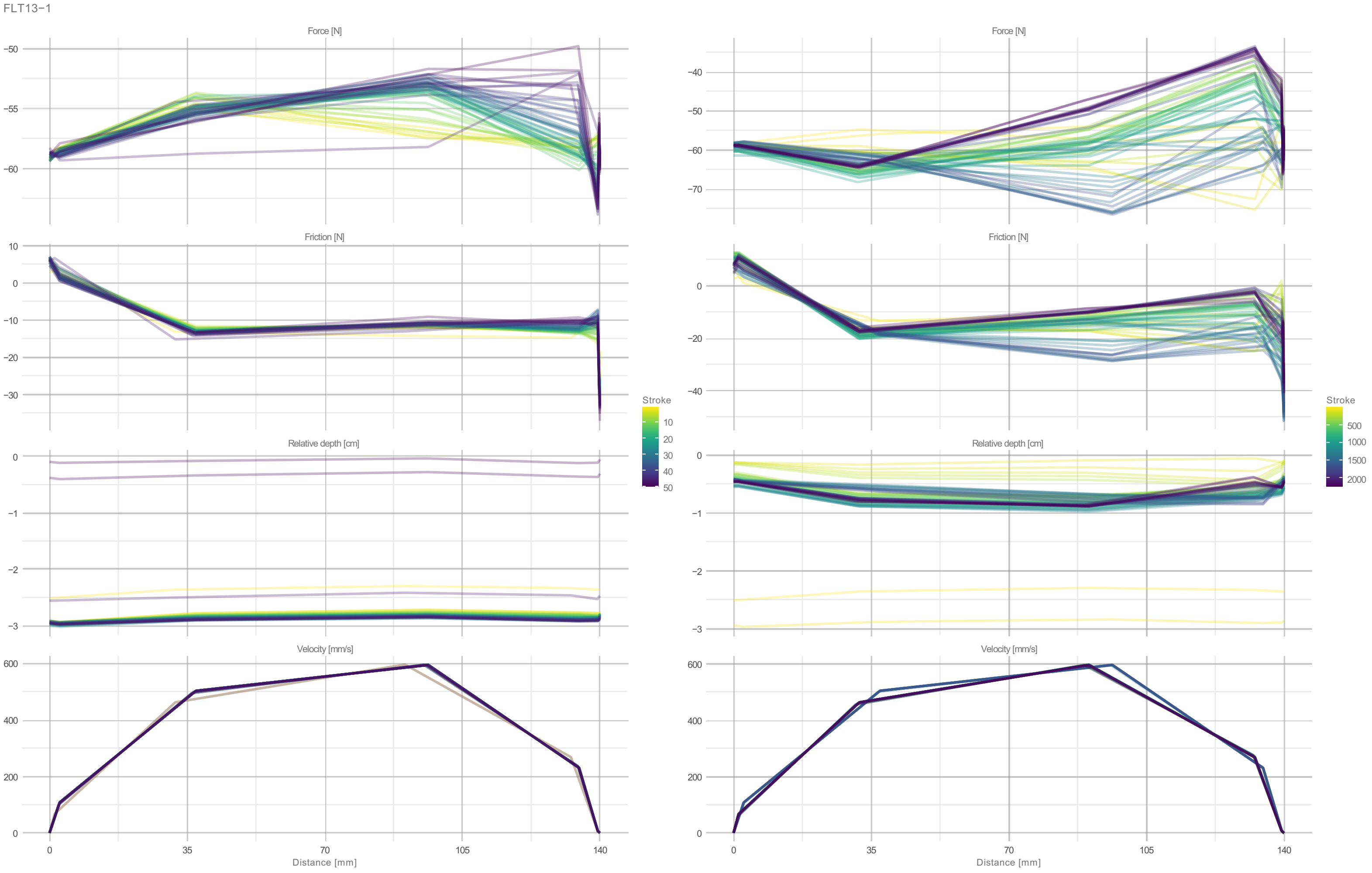

Sensor data (force, friction, relative depth and velocity) of unilinear strokes performed with knapped sample FLT13-1 on bone plate OFA-BP1. Sensor data is illustrated on the y-axis. Distance on the x-axis illustrates the position of the sample on the contact material from the origin of each stroke. The left block of graphs shows the first 50 strokes of the experiment. The right block of graphs shows all 2000 strokes. Lines illustrate every 40th cutting stroke from yellow to purple.

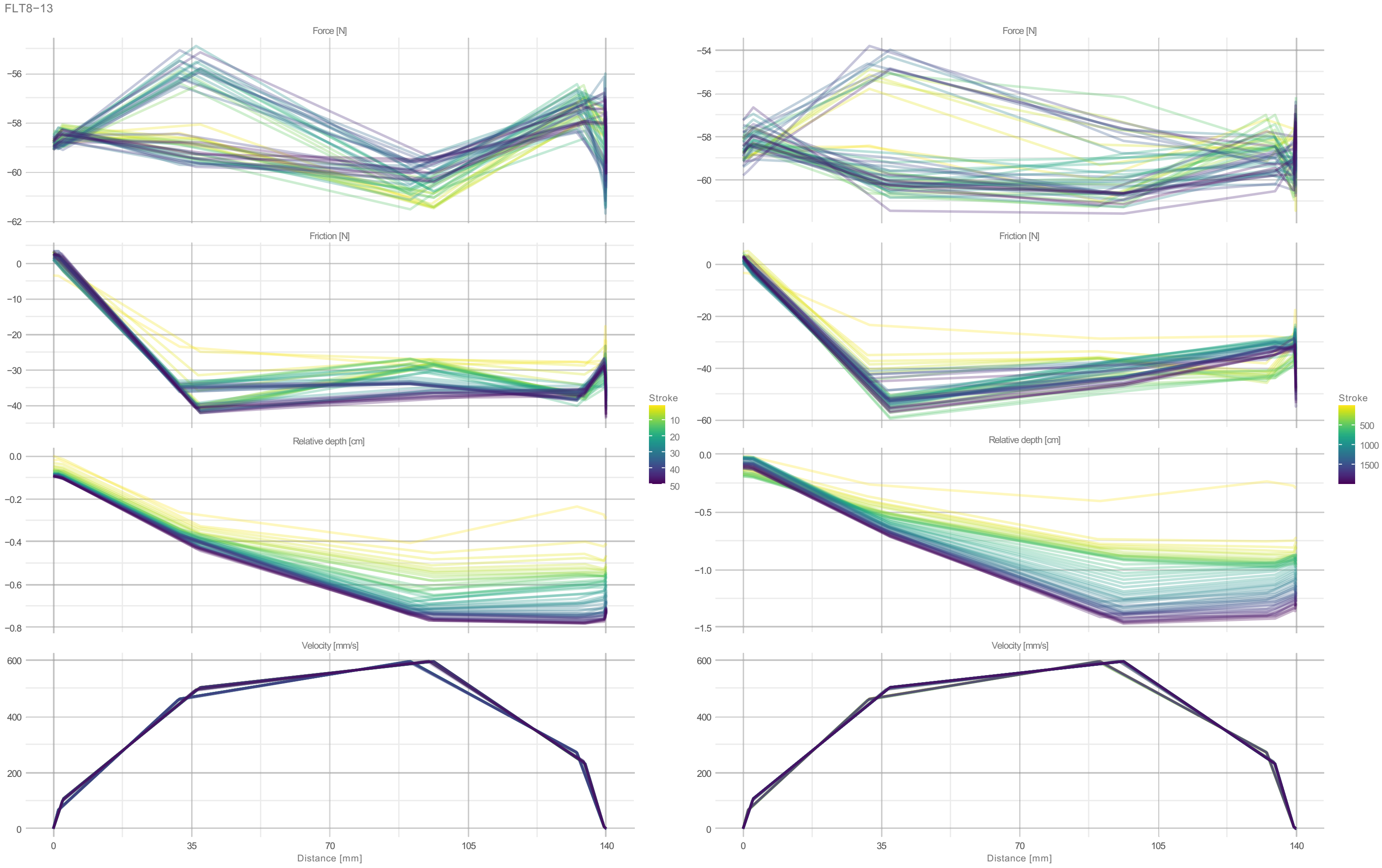

Sensor data (force, friction, relative depth and velocity) of unilinear strokes performed with standard cut sample FLT8-13 on wood plate OFA-WP1. Sensor data is illustrated on the y-axis. Distance on the x-axis illustrates the position of the sample on the contact material from the origin of each stroke. The left block of graphs shows the first 50 strokes of the experiment. The right block of graphs shows all 2000 strokes. Lines illustrate every 40th cutting stroke from yellow to purple.

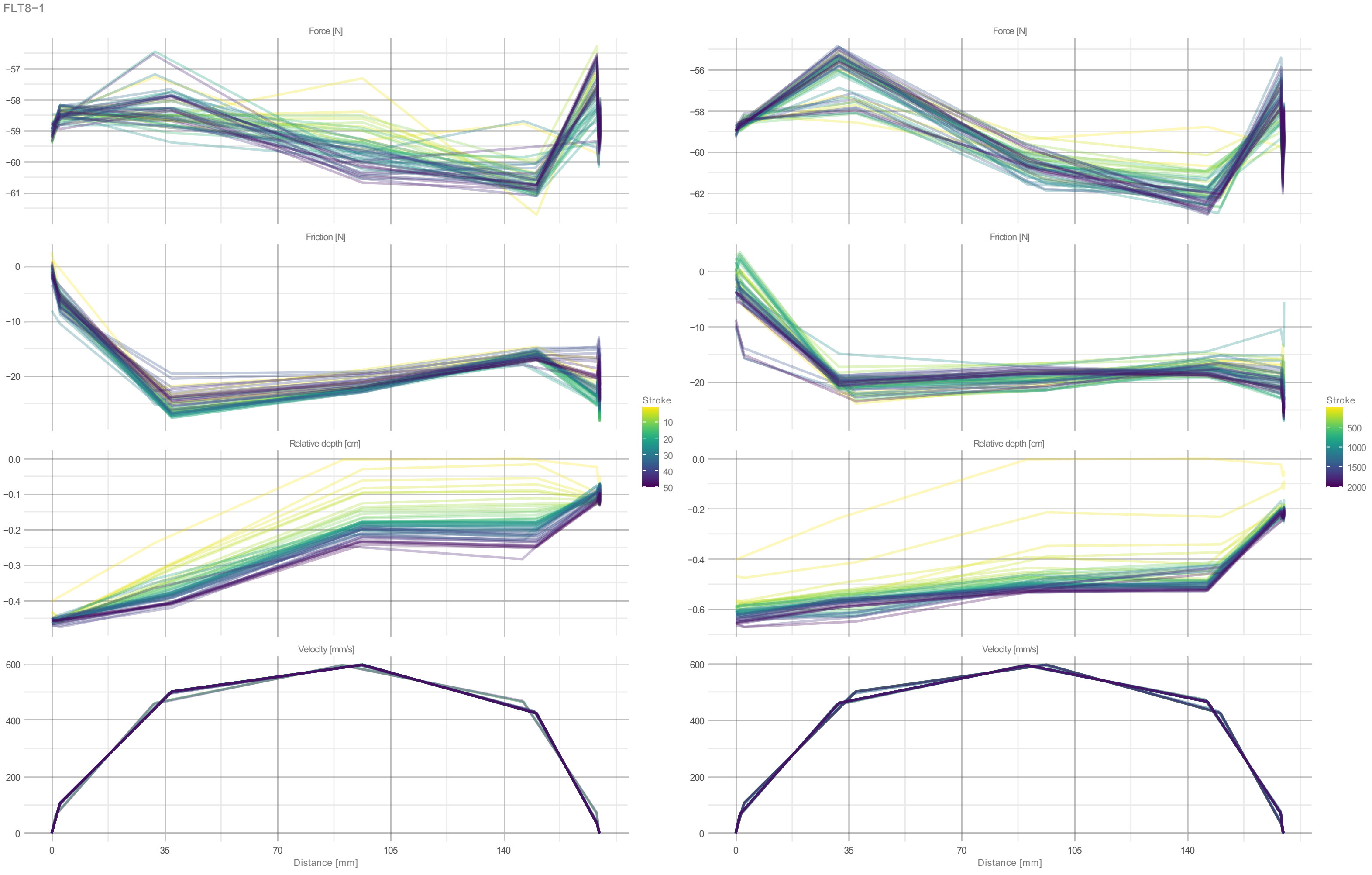

Sensor data (force, friction, relative depth and velocity) of unilinear strokes performed with standard cut sample FLT8-1 on bone plate BP-cutting. Sensor data is illustrated on the y-axis. Distance on the x-axis illustrates the position of the sample on the contact material from the origin of each stroke. The left block of graphs shows the first 50 strokes of the experiment. The right block of graphs shows all 2000 strokes. Lines illustrate every 40th cutting stroke from yellow to purple.

		3D scanner	Digital microscope	Upright light microscope
**Microscope**	Manufacturer	AICON/ Hexagon AB	Carl Zeiss Microscopy GmbH	Carl Zeiss Microscopy GmbH
	Model	smartSCAN-HE R8	Smartzoom 5	Axio Imager.Z2 Vario
**Location**	Laboratory	IMPALA, MONREPOS, Germany	IMPALA , MONREPOS, Germany	IMPALA , MONREPOS, Germany
	Floor	Basement (-1)	Basement (-1)	Basement (-1)
	Setup	Column stand	Stable table on solid concrete base	Passive anti-vibration table on solid concrete base
**Acquisition**	Software	OptoCat 2020R2	Smartzoom software with Shuttle&Find module V1.4 HF7	ZEN blue 2.6 HF 12 with Shuttle & Find module
	Mode	Structured light	Bright field	Bright field
**Objective**	Manufacturer	Schneider-Kreuznach	Carl Zeiss Microscopy GmbH	Carl Zeiss Microscopy GmbH
	Objectives	S-150, 33 µm point-to-point distance	PlanApo D 1.6x / NA= 0.1 / WD= 36 mm	C Epiplan-Apochromat 5x / NA= 0.20 / WD= 21.0 mm
				C Epiplan-Apochromat 10x / NA= 0.40 / WD= 5.4 mm
				C Epiplan-Apochromat 20 x / NA= 0.70 / WD= 1.30 mm
**Illumination**	Source	Blue LED	White LED, reflected ringlight	White LED, reflected light
**Size**	Step size for z-stacks	-	Continuous mode (no steps)	9.19 µm
	FOV per scan/tile	110 x 80 x 70 mm measuring volume	10.475 x 7.856 mm	1702.0 x 1420.2 µm (5x obj.)/ 850.8 x 709.9 µm (10x obj.)/ 426.7 x 356.1 µm (20x obj)
	Frame size	-	1600 x 1200 pixels per tile	2048 x 2048 pixels

Acquisition settings of imaging equipment. NA= numerical aperture, WD= working distance, FOV= field of view.
